# *Helicobacter pylori* infection induces DNA double-strand breaks through the ACVR1/IRF3/POLD1 signaling axis to drive gastric tumorigenesis

**DOI:** 10.1080/19490976.2025.2463581

**Published:** 2025-02-09

**Authors:** Xinbo Xu, Xiao Fei, Huan Wang, Xidong Wu, Yuan Zhan, Xin Li, Yan’an Zhou, Chunxi Shu, Cong He, Yi Hu, Jianping Liu, Nonghua Lv, Nianshuang Li, Yin Zhu

**Affiliations:** a Department of Gastroenterology, Jiangxi Provincial Key Laboratory of Digestive Diseases, Jiangxi Clinical Research Center for Gastroenterology, Digestive Disease Hospital, The First Affiliated Hospital, Jiangxi Medical College, Nanchang University, Nanchang, Jiangxi, China; b Department of Drug Safety Evaluation, Jiangxi Testing Center of Medical Instruments, Nanchang, China; c Department of Pathology, The First Affiliated Hospital, Jiangxi Medical College, Nanchang University, Nanchang, China

**Keywords:** *H. pylori*, ACVR1, POLD1, IRF3, DNA damage

## Abstract

*Helicobacter pylori* (*H. pylori*) infection plays a pivotal role in gastric carcinogenesis through inflammation-related mechanisms. Activin A receptor type I (ACVR1), known for encoding the type I receptor for bone morphogenetic proteins (BMPs), has been identified as a cancer diver gene across various tumors. However, the specific role of AVCR1 in *H. pylori*-induced gastric tumorigenesis remains incompletely understood. We conducted a comprehensive analysis of the clinical relevance of ACVR1 by integrating data from public databases and our local collection of human gastric tissues. In vitro cell cultures, patient-derived gastric organoids, and transgenic INS-GAS mouse models were used for Western blot, qRT-PCR, immunofluorescence, immunohistochemistry, luciferase assays, ChIP, and comet assays. Furthermore, to investigate the therapeutic potential, we utilized the ACVR1 inhibitor DM3189 in our in vivo studies. *H. pylori* infection led to increased expression of ACVR1 in gastric epithelial cells, gastric organoid and gastric mucosa of INS-GAS mice. ACVR1 activation led to DNA double-strand break (DSB) accumulation by inhibiting POLD1, a crucial DNA repair enzyme. The activation of POLD1 was facilitated by the transcription factor IRF3, with identified binding sites. Additionally, treatment with the ACVR1 inhibitor DM3189 significantly ameliorated *H. pylori*-induced gastric pathology and reduced DNA damage in INS-GAS mice. Immunohistochemistry analysis showed elevated levels of ACVR1 in *H. pylori*-positive gastritis tissues, showing a negative correlation with POLD1 expression. This study uncovers a novel signaling axis of AVCR1/IRF3/POLD1 in the pathogenesis of *H. pylori* infection. The upregulation of ACVR1 and the suppression of POLD1 upon *H. pylori* infection establish a connection between the infection, genomic instability, and the development of gastric carcinogenesis.

## GRAPHICAL ABSTRACT

**Figure:**
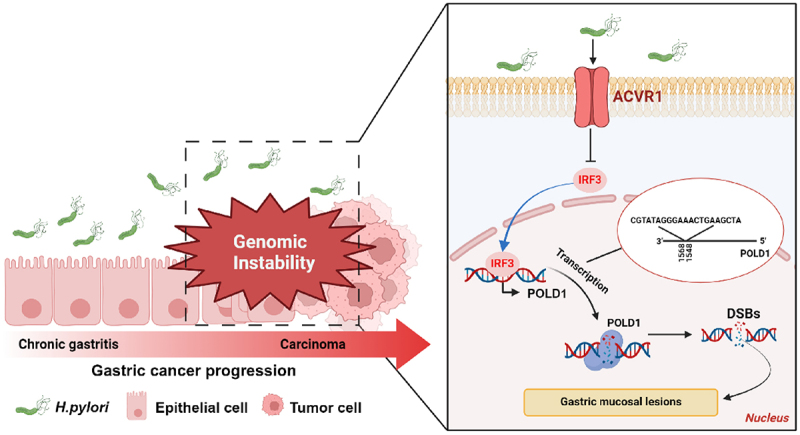

## Introduction

Gastric cancer ranks as the fifth most prevalent cancer and the fourth leading cause of cancer-related death worldwide, with over one million new cases reported annually.^1^ *Helicobacter pylori* (*H. pylori*) affects more than half of the global population.^2,3^ As a class I carcinogen, *H. pylori* infection acts as a “trigger” in the initiation of gastric mucosal carcinogenesis, advancing through chronic non-atrophic gastritis, atrophic gastritis, intestinal metaplasia, dysplasia, and culminating in gastric cancer.^4,5^ *H. pylori* infection directly activates inflammatory responses in gastric epithelial cells by inducting oxidative stress, promoting DNA damage, inhibiting DNA repair pathways, and triggering ER stress.^6–9^ Moreover, chronic inflammation exacerbates DNA damage and hastens the transition to gastric cancer. Additionally, following the development of intestinal metaplasia in the gastric mucosa, even post-eradication of *H. pylori*, the persistent effects of the activation cascade in the gastric mucosal tissue endure, leading to irreversible alterations beyond a return to normalcy.^6^ Thus, comprehending the molecular mechanisms responsible for DNA damage induced by *H. pylori* infection is crucial for advancing strategies in the prevention, diagnosis, and treatment of *H. pylori*-related gastric diseases.

Double-strand breaks (DSBs) in DNA represent one of the most severe forms of damage to the eukaryotic genome, posing a serious threat to normal cellular functions and overall survival.^10^ Reactive oxygen species (ROS), ionizing radiation (IR), external stimuli, and various other methods can all contribute to the induction of DSBs.^11^ Following the occurrence of double-strand breaks (DSBs), organisms trigger a cascade of defense responses known as the DNA damage response (DDR). Following DDR activation, sensor molecules such as ATM and ATR are promptly activated to initiate diverse repair pathways. This process aims to maintain the balance between DNA damage and repair by effectively restoring the integrity of the damaged DNA. Homologous recombination (HR repair) and non-homologous end joining (NHRJ repair) stand out as the two predominant mechanisms utilized for repairing DSBs.^12^ POLD1, situated in the nucleus, encodes the catalytic subunit p125 of DNA polymerase. This molecule plays a pivotal role in HR repair.^13,14^ Upon the occurrence of a double-strand break (DSB), POLD1 is summoned by ATR and moves to the sites of DNA damage. It plays a crucial role in orchestrating the synthesis of the lagging strand and facilitating the subsequent repair of DNA double helix.^15,16^ Recent studies, along with our prior data, have confirmed that *H. pylori* infection can induce DSBs in the early stages and activate the DDR system to repair the damaged DNA.^17–20^ Prolonged *H. pylori* infection has been shown to diminish the expression of Rad51, a key factor for homologous recombination repair, impeding the DNA repair process. This inhibition leads to the accumulation of DNA damage, induction of genomic instability, and advancement of gastric mucosal lesions. However, it remains uncertain whether *H. pylori* infection hampers DSBs repair through alternative mechanisms. Hence, uncovering the pathways by which *H. pylori* influences DNA damage is essential for gaining deeper insights into its involvement in gastric mucosal lesions.

IRF3, a constituent of the interferon regulatory factor (IRF) family, is instrumental in regulating the expression of interferon genes in response to viral infections. Upon activation of the cGAS-STING signaling pathway by viral stimuli, IRF3 is translocated to the nucleus. This event triggers the transcription of downstream molecules such as IFNs and ISGs, which elicits robust antiviral effects.^21,22^ Furthermore, research has highlighted the preventive function of IRF3 in viral inflammation, colorectal tumor progression, and the regulation of cell death, inflammatory reactions, and fibrosis.^23–25^ Nonetheless, the specific association between IRF3 and gastric cancer, particularly concerning *H. pylori* infection, remains inadequately elucidated.

ACVR1 encodes a protein consisting of 509 amino acids and is widely expressed in various tissues and cell types.^26^ As a vital member of the Activin receptor family, ACVR1 plays a pivotal role in cellular growth and development processes. It is located on the cell membrane and governs regulatory functions by triggering the BMP-SMAD signaling pathway.^27^ In various diseases, notably in tumors, ACVR1 commonly acts as a “promoter” of carcinogenesis. Its upregulation has been linked to increased cancer cell growth and proliferation.^27,28^ A study has established an association between ACVR1 and gastric cancer. In our previous study, we discovered that ACVR1 not only correlates with gastric cancer but also fosters the advancement of gastric cancer induced by *H. pylori*.^28,29^

Based on our previous work, through the analysis of transcriptomic data from ACVR1 knockout cells, we observed an activation of HR repair following ACVR1 knockout. This was accompanied by an increase in the expression of DNA polymerase 1 (POLD1), a key player in DNA repair processes. Furthermore, our findings suggest that *H. pylori* infection can suppress the expression of POLD1, potentially representing a novel mechanism through which *H. pylori* induces DSBs.^30^ Accordingly, we hypothesized that the DSBs induced by *H. pylori* infection might be associated with the activation of ACVR1 and the suppression of POLD1. Our study demonstrated, through both *in vitro* and *in vivo* experiments, that *H. pylori* infection induces DSBs by elevating the expression of ACVR1. Our research unveiled that the double-strand breaks (DSBs) induced by ACVR1 are dependent on the suppression of POLD1. We further explored the precise mechanism through which ACVR1 hampers the transcription of POLD1 via IRF3, using transcription factor prediction methods. Ultimately, our investigations revealed that administering ACVR1 inhibitors ameliorated *H. pylori*-induced gastric mucosal injuries in mice.

In conclusion, this study provides preliminary insights into the involvement of ACVR1 in modulating DSB triggered by *H. pylori* infection. Our results shed light on a novel mechanism through which *H. pylori* infection stimulates the expression of ACVR1, fostering DNA damage and the development of gastric tumorigenesis. These findings hold promise in identifying therapeutic targets for the prevention and treatment of *H. pylori*-related gastric mucosal disorders.

## Materials and methods

### H. pylori *strains and cell lines*

The wild-type *H. pylori* strains PMSS1, NCTC11637 and 26,695 were used in this study. Briefly, *H. pylori* bacteria were cultured on Brucella agar supplemented with 5% sheep blood (BD Bioscience) under microaerophilic conditions for *in vitro* passages, following established protocols.^17,31^ For *in vitro* co-culture with gastric epithelial cells, the bacteria were then propagated in Brucella broth (BD Bioscience) supplemented with 10% FBS (BD Bioscience) overnight. *H. pylori* PMSS1 and NCTC11637 strains were co-cultured with gastric epithelial cells at a multiplicity of infection (MOI) of 100:1. Additionally, the PMSS1 strain was utilized in experiments involving mice.

The human gastric cancer cell AGS and epithelial cell HFE145 were cultured in DMEM/F12 and DMEM medium, respectively, supplemented with 10% FBS (BD Bioscience) and 1% penicillin/streptomycin (NCM Biotech). Both cell lines were maintained at 37°C in a humidified atmosphere of 5% CO_2_.

### Patients and tissue samples

The gastritis tissues were obtained from The First Affiliated Hospital of Nanchang University, and categorized into two groups: *H. pylori*-positive (*n* = 20) and *H. pylori*-negative (*n* = 20). The *H. pylori*-positive group comprised patients who were newly identified as *H. pylori*-positive for the first time. Individuals with a prior history of *H. pylori* eradication or other infectious diseases were excluded from the study. The study protocol and the waiver of informed consent were authorized by the Ethics Committee of The First Affiliated Hospital of Nanchang University. The presence of *H. pylori* infection was ascertained using the carbon-13 urea breath test.

### Human gastric organoid culture

Human gastric cancer tissues were procured from The First Affiliated Hospital of Nanchang University. To culture human gastric organoids, we followed the protocol previously published.^32^ The gastric organoids were dissociated using bioGenous^TM^ Gastric Cancer Organoid Kit (K2179-GC, BioGenous Technologies). Briefly, the tumor tissue was sliced into 1–2 mm pieces followed by digestion with 10 ml of Tissue Digestion Solution (K601003) at 37°C for 30 min. Following this, FBS was added to the tissue digestion mixture to achieve a final concentration of 2% and the mixture was filtered using a 100 µm cell strainer. The filtered cells were harvested and centrifuged at 250 g for 3 min at 4°C. After removing the supernatant, the pellet was resuspended in Epithelial Organoid Basal Medium (B213152) and centrifuged at 250 g for 3 min at 4°C.

Following the previous steps, aspirate the supernatant and resuspend the pellet in Organoid Culture ECM (M315066 BioGenous Technologies). Place the ECM-containing organoids on the bottom of 24-well cell culture plates in droplets of 30 µL each around the center of the well. After the ECM droplets solidify (15–25 min), carefully add to each well 500 µL of Organoid Complete Medium: Advanced DMEM/F12 Medium (Gibco) supplemented with 50% Wnt3a and 25% R-spondin conditional medium, 100 ng/mL human Noggin (Peprotech, NJ, USA), 1% B27 (Invitrogen, CA, USA), 1% *N*-2 (Invitrogen), 4 mm Nicotinamide (Sigma-Aldrich), 200 ng/mL human fibroblast growth factor-10 (FGF-10, Peprotech), 50 ng/mL epidermal growth factor (EGF, Invitrogen), 1 mm N-Acetyl-Lcysteine (Sigma-Aldrich), 1 nM Gastrin (Tocris, MN, USA), 0.5 μM A83–01(Tocris), 10 μM Y-27632(Sigma-Aldrich). The gastric organoids were cultured in 5% CO_2_ incubator at 37°C, with the media changed twice a week. To generate gastric organoids post-*H. pylori* infection, fully developed gastric organoids were dissociated into 15 ml tubes with culture media, followed by a 12 h infection with *H. pylori* or 24 h transfection with siRNA. Immunofluorescence staining using an *H. pylori* antibody (ab7788, abcam) was performed to visualize *H. pylori* colonization.

### *Infection with* H. pylori *in animals*

All procedures performed on animals were approved by the Ethics Committee of First Affiliated Hospital of Nanchang University. The transgenic insulin-gastrin (INS-GAS) mouse models, extensively utilized for investigating the pathogenesis of *H. pylori*, was employed as previously described.^33^ Male specific-pathogen-free INS-GAS mice (Jackson Lab) aged between 6 and 8 weeks were housed under a 12 h light/dark cycle and provided *ad libitum* access to food and water. After 1 week of acclimation, the mice were gavage with the mouse-adapted wild-type *H. pylori* strain PMSS1 (2 × 10^9^ CFU/mouse) every other day for eight times, or were given Brucella Broth as an uninfected control group. The mice were sacrificed after 4 months of infection for further analyses.

### Treatment with ACVR1 inhibitor in mice

The ACVR1 inhibitor DM3189 was procured from Selleck, China (S2618). The INS-GAS mice were randomly allocated into two groups: the *H. pylori*-infected group and the *H. pylori* combined with DM3189 treatment group. DM3189 were dissolved in PBS. Following 4 weeks of *H. pylori* infection, the mice received gavage administration of 3 mg/kg DM3189 for a duration of 10 weeks. Subsequently, all mice were sacrificed at 13 weeks post infection for analyses.

### Transfection with plasmids or siRNAs

All transfections were carried out in Opti-MEM (Gibco, USA) using Lipofectamine 3000 following the manufacturer’s instruction (L3000015, ThermoFisher, USA). The overexpression plasmids for POLD1, ACVR1, IRF3 (purchased from Hitro BioTech, Beijing, China), along with the relevant siRNA (purchased from GenePharma, Suzhou, China) were transfected into cells in 6-well plates. Following a 24-h transfection period, the cells were co-cultured with *H. pylori* (MOI = 100) for 24 h. Subsequently, the cells were harvested 48 h post-transfection with plasmids and siRNA for further analyses.

### *Evaluation of* H. pylori *colonization in animal models*

*H. pylori* colonization in mouse gastric tissues was evaluated through silver staining and colony-forming assays. The silver staining was performed using the Spirochete Silver Staining Kit (Warthin–Starry Method) (Solarbio-G1940). Initially, the paraffin-embedded tissues were deparaffinized using xylene and ethanol gradient. Subsequently, the slides were immersed in the Acid Sliver Solution for staining in the water bath at 56°C for 1 h. Following this step, the slides were re-stained with Warthin-Starry Stain Solution in water bath at 56°C until they appeared yellowish brown. All images were acquired under a Nikon Eclipse microscope.

In the colony-forming assay, the murine stomachs were dissected along the inner curvature and sectioned into four parts. One quarter of the stomach was homogenized in brain heart infusion (BHI) after weighing. Subsequently, gradient dilutions were prepared in BHI broth and portions of the dilutions were spread onto Brucella agar plates. Following a 2-day culture, colonies were enumerated, and the colony-forming units per stomach was calculated. The colonies were verified to be *H. pylori* through Gram staining.

### Western blotting assay

For immunoblotting, cells and tissues were lysed at 4°C for 30 min in Lysis Reagent (SolarbioR0020, China) supplemented with a cocktail of phosphate and proteinase inhibitors. The lysates were then separated by 10% SDS polyacrylamide gel electrophoresis (PAGE) and transferred to nitrocellulose membrane (PerkinElmer, Waltham, MA). Subsequent to blocking with 5% BSA in PBS, the membrane was sequentially incubated with primary antibodies at 4°C overnight, and with the corresponding secondary antibodies at room temperature for 1 h. Visualization was achieved using the BIO-RAD ChemiDoc XRS + system. The antibodies used in the study were as follows: ACVR1(ab155981), POLD1 (ab186407), IRF3(ab68481) and p-IRF3(S386) (EPR2346) antibodies were purchased from abcam (USA), while β-actin (#4967), γ-H2AX (#80312), ATM(#2873), p-ATM (#5883), Chk2 (2662), p-Chk2(#2661) and GAPDH(D16H11) antibodies were obtained from Cell Signaling Technology (USA).

### Total RNA extraction and quantitative Real-Time PCR (qRT-PCR) assay

TRIzol (Invitrogen, Carlsbad, CA, USA) was used to extract total RNA from tissues or cells. First, the cells or homogenized samples were incubated with 1 ml of TRIzol to allow complete dissociation of nucleoprotein complexes. Then, 0.2 ml chloroform per ml of TRIzol was added, followed by a vigorous mixing for 15 sec and a centrifuge at 10,000 g for 10 min at 4°C to facilitate phase separation. Next, the upper clear phase was carefully transferred to a fresh tube, followed by an addition of 0.4 ml isopropanol per each milliliter of the clear phase. Subsequently, the precipitated RNA was collected by centrifugation at 10,000 g for 10 min at 4°C. Finally, the RNA precipitate was wash ed with ethanol.

For reverse transcription of RNA into cDNA, the FastKing RT Kit (KR116, TIANGEN, Biotech, China) was used. The mRNA levels were quantified using SYBR Green dye and PCR master mix (YEASEN, Shanghai, China) with QuantStudio 5 Real-Time PCR system. Gene-specific primers (Suplementary Table 1) were obtained from the public database PrimerBank (https://pga.mgh.harvard.edu/primerbank/). The results presented were from three independent experiments performed in triplicate.

### Immunofluorescence assay

Cells grown on the coverslips were washed with ice-cold PBS, fixed with 4% formaldehyde in PBS for 30 min, and then permeabilized with 0.5% Triton-100 at room temperature for 10 min. After blocking with 3% bovine serum albumin (BSA) in PBS for 1 h, the cells were incubated with primary antibodies at 4°C for overnight. The following antibodies and dilutions were used anti-Phospho-Histone (Ser139) Mouse mAb (D7T2V, CST: 1:100), anti-POLD1 rabbit monoclonal (ab186407, abcam: 1:200), anti-ACVR1 mouse monoclonal (2C1E8, Invitrogen: 1:100), anti-IRF3 mouse monoclonal (D9J5Q, CST: 1:200), anti-β-catenin mouse monoclonal (15B8, CST: 1:100), anti-MUC5AC mouse monoclonal (45M1, Invitrogen: 1:100). Coverslips were washed with PBS for three times and incubated with the corresponding secondary antibody conjugated with Alexa Fluor Plus 488 or Alexa Fluor Plus 555 (1:500 dilution, Thermo Fisher, Weston, FL) in the dark for 1 h. Cell nuclei were counterstained with 4′,6-diamidino-2-phenylindole (DAPI). Images were acquired with a Leica Stellaris 5 fluorescent microscope using a 63×/1.4 NA objective with an oil immersion. For the analysis of γ-H2AX foci, automated image acquisition was performed on IN Cell Analyzer 2200 (General Electric Company, USA) using a 40×/0.9 NA objective. The analysis of acquired images, commonly referred to as a quantitative image-based cytometry, was performed using IN Cell Analyzer 2200 3.7.1.(Jiangxi Provincial Key Laboratory of Digestive Diseases, Nan Chang, CHINA)

### Histopathology and immunohistochemistry staining

The tissue specimens were initially fixed in a 4% paraformaldehyde solution (P1110, Solarbio, China) overnight at 25°C. Then, the samples were processed using an automated tissue dehydrator (ASP300S; Leica Biosystems, USA). Next, the organs were embedded in paraffin using an embedding machine (EG1150®; Leica Biosystems). Tissue sections of 4 μm thickness were sliced using a microtome (RM2235, Leica Biosystems) and mounted on glass slides (188105, Citotest, China).

For pathological tissue staining, the sections were initially deparaffinized and rehydrated. Then, the slides were stained with hematoxylin (L25050202, YULU, China) for 3 min and washed with tap water, followed by staining with eosin (L25050403, YULU, China) for 30 s and re-washing with tap water. Finally, the slides were dehydrated and mounted medium for image acquisition under a Nikon microscope.

In the immunohistochemical staining (IHC) procedures, following deparaffinization and rehydration of tissue sections, a 30-min incubation in a 1% BSA blocking solution was employed to minimize nonspecific antibody binding. Subsequently, overnight exposure to the respective primary antibodies at 4°C ensued. Post-primary antibody incubation, PBS washing was carried out, succeeded by a 1-h incubation at 37°C with the secondary antibody (PV-6000, ZSGB-BIO, China). Color development was achieved using DAB (ZLI-9017, ZSGB-BIO, China), followed by counterstaining with hematoxylin. Stained sections were examined using a light microscope (Olympus IX 70, Tokyo, Japan) for image acquisition.

### Luciferase assay

The luciferase promoter plasmid, encompassing a DNA sequence spanning from −2000 bp upstream of the translation start site to 100 bp beyond translation start site of POLD1 gene, was provided by Hitro Biotech (Beijing, China). Transfection of the plasmids was conducted utilizing Lipofectamine 3000 following the manufacturer’s instruction (L3000015, ThermoFisher, USA). Subsequent to a 48-h incubation period, the cells were exposed to *H. pylori* (PMSS1, NCTC11637) for 8 h. The Dual Luciferase Reporter Gene Assay Kit (Yeasen Biotechnology shanghai, China) were employed for downstream analyses.

### Cellular ROS detection and N-acetyl-cysteine (NAC) treatment

For intracellular ROS detection, cells were washed with PBS and exposed to a 10 μM Reactive Oxygen Species Assay Kit (50101ES01, TEASEN, CHINA) at 37°C for 30 min in the dark for intracellular ROS detection. Intracellular levels of emitted fluorescence were observed under a fluorescence microscope after thorough washing.

For NAC treatment, cells were co-cultured with *H. pylori* and treated with 5 mm NAC (S1623, Selleck, USA) for 24 h. Subsequently, cells were collected for protein expression analysis and ROS detection.

### Single-cell gel electrophoresis/comet assay

AGS and HFE145 cells were subjected to transfection with either knockdown siRNA or overexpression plasmids. Subsequently, cells were plated at a density of 2 × 10^5^ cells per well in six-well plates. The comet assay was conducted as previously outlined.^17^ Briefly, cells were embedded in low-melting-point agarose (A8350 Solarbio, Beijing, China) and spread on glass slides. The slides were immersed in lysis buffer at 4°C for 1 h and then exposed to neutral electrophoresis buffer for 60 min to facilitate DNA unwinding. Electrophoresis was carried out at 30 V for 30 min. Comet tails were visualized by staining with propidium iodide (PI). Quantitatively, comets on each gel were examined using a fluorescence microscope, with tail length and tail moment serving as metrics to assess the extent of DNA damage.

### ChIP assay

The Simple ChIP® Enzymatic Chromatin IP Kit (Magnetic Beads) (#9003, Cell Signaling Technology, USA) was used, following the manufacturer's protocol. AGS or HFE145 cells were cultured in a 15 cm plate, 1 day before treatment with *H. pylori* PMSS1 for 8 h. Cross-linking of proteins with DNA was achieved using formaldehyde. Then, a cocktail of enzymes provided by the kit was used to fragment DNA at 37°C for 15 min. The IRF3 and IgG antibodies were added to the chromatin along with Protein G magnetic beads overnight to form the ChIP and input complexes. Chromatin was eluted from the magnetic beads and proteinase K was added to reverse the cross-link ing process and extract the DNA. Quantification of the amplified DNA fragments was carried out through a qRT-PCR assay, with primer sequences detailed in Supplementary Table 1.

### Statistical analysis

All statistical analysis was performed using GraphPad prism 9.0 software. Data were presented as mean±SD derived from three independent experiments. Student’s t test was applied for comparisons between two groups, while one-way analysis of variance (ANOVA) was used for comparisons involving more than two groups. Pearson correlation analysis was employed to assess the correlation between ACVR1, POLD1, and γ-H2AX expression levels. The significance levels are denoted as follows: *p < 0.05; **P  < 0.01; ***p < 0.005.

## Results

### H. Pylori *infection induced an upregulation of ACVR1 expression in gastric epithelial cells, gastric organoids, and transgenic mouse model*

**Figure 1.**
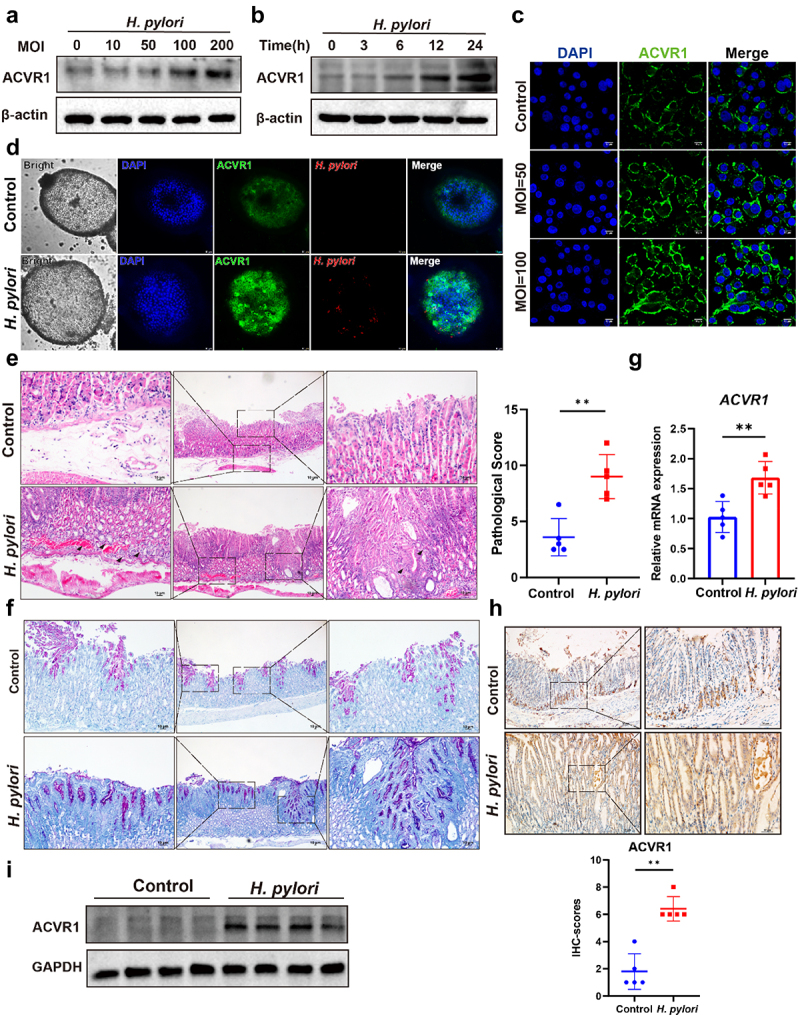
*H. pylori* infection induced ACVR1 expression in gastric epithelial cells, gastric organoids and transgenic mouse model. (a and b) Western blot analysis of ACVR1 expression in AGS cells following *H. pylori*PMSS1 infection with different MOIs (multiplicity of infection) (0,10,50,100,200) (a) and infection time (0 h, 3 h, 6 h, 12 h, 24 h,) (b). (c) Immunofluorescence staining to determine the ACVR1 cellular localization in AGS cells following *H. pylori*PMSS1 infection with different MOI for 24 h. (Scale bar: 10 µm) (d) Immunofluorescence staining for ACVR1 and *H. pylori* in gastric organoid infected with the *H. pylori* PMSS1 strain for 24 h (Scale bar: 10 µm). (e and f) INS-GAS mice were infected with *H. pylori* PMSS1 strain for 4 months. H&E (e) and AB-PAS (f) staining of representative histological features of gastric mucosa from different groups. The histopathological features of gastric mucosa for all mice were analyzed. (Scale bar: 10 µm, *n* = 5 mice) (g) qRT-pcr of ACVR1 expression in *H. pylori* infected mice, in comparison with control groups (*n* = 5 mice). (h) Representative immunohistochemistry staining and quantification analysis of ACVR1 in stomach tissues from the indicated groups of mice. (Scale bar:10 µm, *n* = 5 mice). (i) Western blot analysis of ACVR1 expression in *H. pylori* infection INS-GAS mice. (*n* = 4 mice). Data are shown as means ± SD. *P* values were calculated using Student’s t test; *, *P* < 0.05; **, *P* < 0.01; ***P < 0.001. Independent experiments were performed a minimum of two times. The data presented are representative results.

In our previous work, we observed elevated ACVR1 expression in gastric cancer, correlating with an unfavorable prognosis in patients with gastric cancer.^28^ It is widely acknowledged that *H. pylori* plays a crucial role in promoting the development of gastric cancer.^6,34^ For a more in-depth exploration into the interplay between ACVR1 and *H. pylori* infection, AGS cells were infected with *H. pylori* strain PMSS1 at varying time points and multiplicities of infection (MOI). Western blot data analyses revealed that *H. pylori* augmented ACVR1 protein expression in a manner dependent on both MOI and exposure duration (Figure 1(a,b)). Furthermore, immunofluorescence staining illustrated a notable enhancement in ACVR1 fluorescence intensity following *H. pylori* infection in AGS cells, as evidenced by the quantification of fluorescence intensity in at least 100 cells (Figure 1(c), Figure S1, A).

The impact of *H. pylori* on ACVR1 expression was further investigated using an *in vitro* gastric organoid system. Immunostaining of basolateral membrane associated protein β-catenin and mucin MUC5AC validated the successful establishment of gastric organoid (Figure S1, B). Consistently, there was a substantial elevation in ACVR1 expression following *H. pylori* infection when compared to non-infected human gastric organoids (Figure 1(d)).

To confirm these observations in an *in vivo* setting, we infected INS-GAS transgenic mice with the *H. pylori* PMSS1 strain, pre-mouse adapted SS1 strain, for a duration of 4 months (Figure S1, C). Silver staining and colony-forming units (CFU) assays verified the colonization of *H. pylori* in gastric mucosa of all infected mice (Figure S1, D and E). As anticipated, *H. pylori*-infected mice developed notable gastric pathological changes in comparison to the uninfected controls (Figure 1(e)). Therefore, we examined alterations in immune responses and inflammation in mice. Immunohistochemistry analysis revealed heightened levels of CD45-positive cells and F4/80-positive cells in the gastric mucosal tissue of mice infected with *H. pylori* (Figure S1, F and G). Meanwhile, the qRT-PCR analysis showed that *H. pylori* infection led to an increase in mRNA levels of proinflammatory cytokines *IL-1β* and *IL-18* (Figure S1, H). Moreover, AB/PAS staining highlighted a substantial presence of blue-stained goblet cells in the gastric tissues of *H. pylori* infected mice (Figure 1(f)). These findings suggest that *H. pylori* infection contributes to the development of gastric mucosal lesions. Next, we determined the expression levels of ACVR1 in stomach tissues from mice. Our data revealed a noticeable elevation in *ACVR1* mRNA levels in *H. pylori*-infected mice compared to the control group (Figure 1(g)). Further affirmation was obtained through immunohistochemistry and western blot analysis, which displayed a significant upregulation of ACVR1 protein levels in the gastric tissues of *H. pylori*-infected mice (Figure 1(h,i)). Overall, these findings convincingly demonstrate that *H. pylori* infection not only triggers lesions in *vitro* and *in vivo* but also leads to an increase in ACVR1 expression.

### ACVR1 downregulated POLD1 expression and induced DSBs

**Figure 2.**
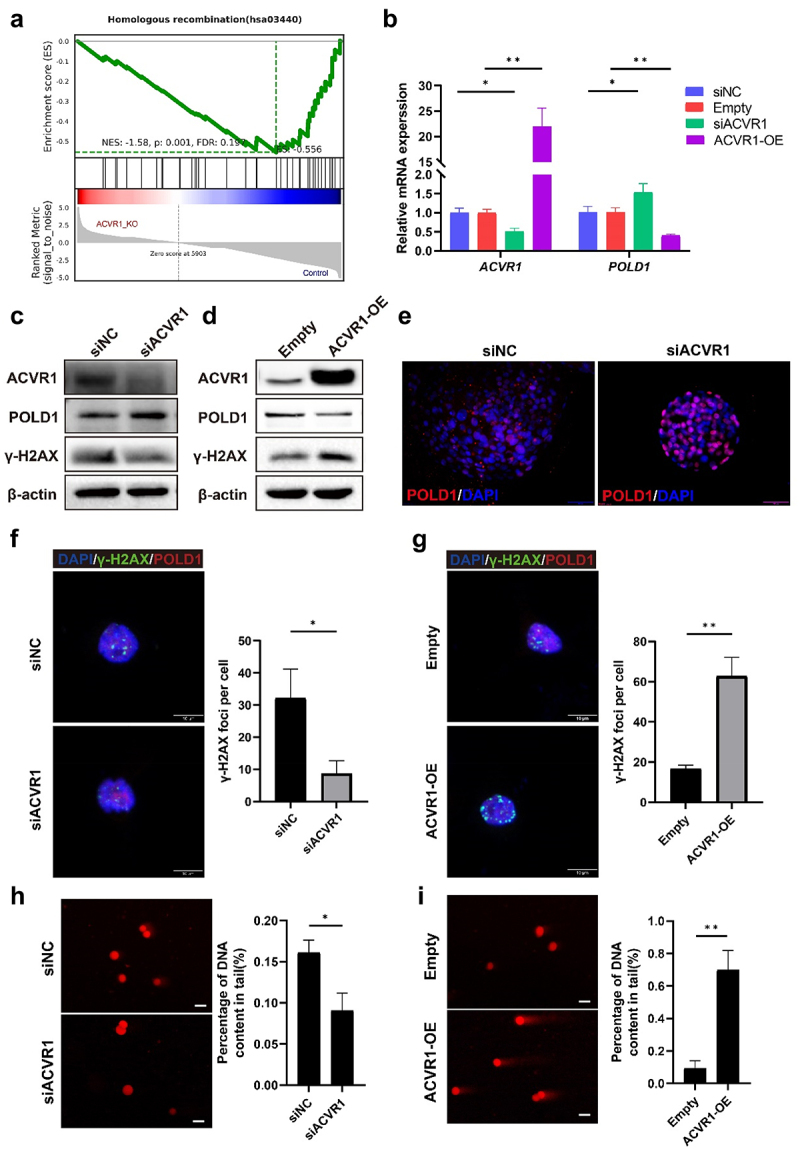
ACVR1 downregulated POLD1 expression and induced DSBs. (a) Transcriptomic analysis using RNA-seq of ACVR1 knockout AGS cells. GSEA analysis reveals alterations in Homologous Recombination repair (HR repair) following ACVR1 knockout. (b to i) AGS cells and gastric organoids were transfected with ACVR1 knockdown siRNA and overexpression plasmids. qRT-pcr analysis showing the mRNA level of POLD1 (b). Western blot analysis showing the protein levels of POLD1, γ-H2AX after ACVR1 knockdown (c) and overexpression (d). Immunofluorescence staining to determine the expression of POLD1 in gastric organoid transfected with siRNA sequence targeting ACVR1 (Scale bar, 50 µm) (e). Representative images of comets, γ-H2AX foci formation are shown in (f to i) (Scale bar:10 µm). Data are representative of at least three independent experiments; ~1000 cells per condition are show in (f and g); ~100 cells per condition are shown in (h and i). Data are shown as means ± SD. *p* values were calculated using Student’s t test; *, *P* < 0.05; **, *P* <0.01; ***P < 0.001.

To investigate the potential contribution of ACVR1 in gastric carcinogenesis, we analyzed the transcriptome data obtained from ACVR1-knockout AGS cell lines as per our previous work.^28^ Gene set enrichment analysis (GSEA) unveiled the activation of homologous recombination (HR) repair pathways following ACVR1 knockout (Figure 2(a)). Further analysis of the differential gene expression within this pathway highlighted a significant upregulation of DNA polymerases, particularly POLD1 and POLD2, in cells with ACVR1 knockout (Figure S2, A). Considering the pivotal role of DNA polymerases in HR repair, we postulated that ACVR1 may impede DNA repair by regulating POLDs, consequently fostering the accumulation of DNA damage.^13,35,36^

To test this hypothesis, we conducted ACVR1 silencing or transient overexpression experiments in gastric epithelial AGS cells. Interestingly, our findings revealed that the upregulation of ACVR1 significantly inhibited POLD1 mRNA and protein levels in both cell line and gastric organoid (Figure 2(b-e)), without impacting POLD2 (Figure S3, A). Conversely, siRNA-mediated ACVR1 knockdown led to an increase in POLD1 expression levels. Remarkably, enhanced ACVR1 expression resulted in a notable elevation of γ-H2AX, whereas γ-H2AX levels decreased upon ACVR1 silencing (Figure 2(c,d)). After the downregulation of ACVR1 in gastric organoids, a substantial increase in nuclear expression of POLD1 was detected (Figure 2(e)). Consistent with these results, transient ACVR1 overexpression inhibited POLD1 and intensified γ-H2AX foci within the cell nucleus, while ACVR1 knockdown exhibited opposing effects (Figure 2(f,g)). Moreover, a substantial increase in comet tail length, indicative of DNA damage, was observed in groups with ACVR1 overexpression (Figure 2(h,i)). Collectively, these results demonstrate that ACVR1 activation inhibits POLD1 expression and induces DSB during *H. pylori* infection.

### H.Pylori *infection suppressed POLD1, subsequently leading to DSBs accumulation*

**Figure 3.**
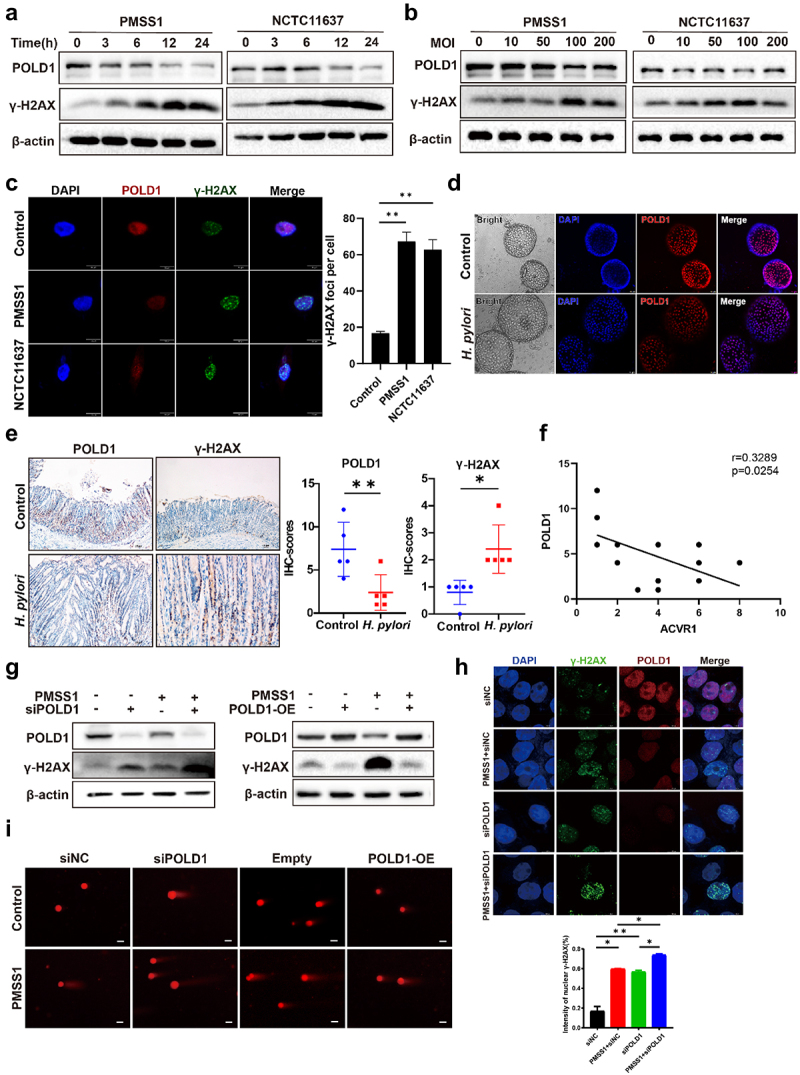
*H. pylori*infection suppressed POLD1, consequently leading to DSBs accumulation. (a and b) Western blot analysis of POLD1 and γ-H2AX expression in AGS cells following infection with *H. pylori*PMSS1 (a) and NCTC11637 (b) for different time duration (0 h, 3 h, 6 h, 12 h. 24 h) and with different MOI (0, 10, 50, 100, 200). (c) Immunofluorescence staining showing the cellular localization of γ-H2AX and POLD1 in AGS cells infected with *H. pylori* PMSS1 and NCTC11637 at 24 h (MOI = 100). (Scale bar:10 µm). (d) Immunofluorescence staining to determine the cellular localization of POLD1 in gastric organoid infected with the *H. pylori* PMSS1 strain (Scale bar,10 µm). (e) Representative immunohistochemistry staining and quantification analysis of POLD1 and γ-H2AX in stomach tissues from the indicated groups of mice. (Scale bar:10 µm) (*n* = 5 mice) (f) Spearman’s correlation between the IHC staining scores of ACVR1 and POLD1 in indicated groups. (*R* = 0.3289, *p* = 0.0254) (*n* = 10 mice). (g to i) AGS cells were transfected with POLD1 knockdown siRNA or overexpression plasmids, and co-cultured with *H. pylori*PMSS1 strain. Western blot analysis showing the expression of POLD1 and γ-H2AX (g). Representative micrographs and quantification data for γ-H2AX foci formation (h). (Scale bar:10 µm). Representative micrographs of comet assay (I) (Scale bar:10 µm). Data are shown as means ± SD. ~1000 cells per condition are show in (c); ~100 cells per condition are shown in (i). *P* values were calculated using Student’s t test; *, *P* < 0.05; **, *P* < 0.01; ***P < 0.001. Independent experiments were performed at least two times. The data presented are representative results.

It is well known that *H. pylori* infection triggers DSBs in gastric epithelial cells.^17,19^ Correspondingly, exposure to *H. pylori* strains PMSS1 and NCTC11637 induced the expression of DSBs marker γ-H2AX and led to an increase in comet tail length (Figure 3(a,b), Figure S3, B). Considering the essential function of POLD1 in DNA damage repair mechanisms, we postulated that *H. pylori* promoted the occurrence of DSBs by downregulating POLD1.

We first investigated the potential of *H. pylori* infection to suppress POLD1 expression. Western blot analyses revealed a significant reduction in both the protein and mRNA levels of POLD1 in a manner dependent on the multiplicity of infection (MOI) and time of exposure to *H. pylori* (Figure 3(a,b) Figure S3, C). Furthermore, immunofluorescence staining illustrated a substantial elevation in γ-H2AX levels and a decrease in nuclear fluorescence intensity of POLD1 following *H. pylori* infection (Figure 3(c)). Similar findings were observed in human gastric organoids, with a decrease in POLD1 expression noted in response to *H. pylori* infection (Figure 3(d), Figure S3, D). Next, we assessed POLD1 and γ-H2AX expression in stomach tissues obtained from *H. pylori*-infected INS-GAS mice. Notably, the infected mice exhibited diminished POLD1 levels in gastric tissues, alongside increased γ-H2AX levels (Figure 3(e)). Spearman’s correlation analysis demonstrated a significant negative correlation between ACVR1 and POLD1 (Figure 3(f) *R* = 0.3289, *p* = 0.0254), as well as a positive correlation between ACVR1 and γ-H2AX (Figure S3, H, *R* = 0.4093, *p* = 0.0102). Furthermore, qRT-PCR and western blot results validated that *H. pylori* significantly suppressed both POLD1 mRNA and protein levels, respectively (Figure S3, E and F). Similar to our previous study, the influence of *H. pylori* on POLD1 and ACVR1 seems to exhibit no significant correlation with the virulence factor VacA (Figure S3, G).

To investigate whether suppression of POLD1 contributes to DNA damage in response to *H. pylori* infection, we examined γ-H2AX expression following POLD1 knockdown or overexpression. Remarkably, POLD1 silencing leads to a striking increase in γ-H2AX expression, whereas POLD1 overexpression resulted in reduced γ-H2AX levels, compared to their respective control groups (Figure S3, I). Furthermore, our results demonstrated that the elevation of DSBs molecular marker γ-H2AX induced by *H. pylori* infection was exacerbated by POLD1 knockdown, while the overexpression of POLD1 abolished *H. pylori*-induced γ-H2AX accumulation (Figure 3(g)). Immunofluorescence staining showed an augmentation in nuclear γ-H2AX foci upon *H. pylori* infection, which was notably intensified upon POLD1 knockdown (Figure 3(h)). Consistent with these observations, the comet assay demonstrated that POLD1 knockdown exacerbated *H. pylori*-induced DNA damage, while POLD1 overexpression mitigated this effect (Figure 3(i) and Figure S3, J). Together, our findings provide compelling evidence that *H. pylori* infection stimulates DSBs by downregulating POLD1.

### H.Pylori *infection promoted POLD1-mediated DSBs accumulation by activating ACVR1*

**Figure 4.**
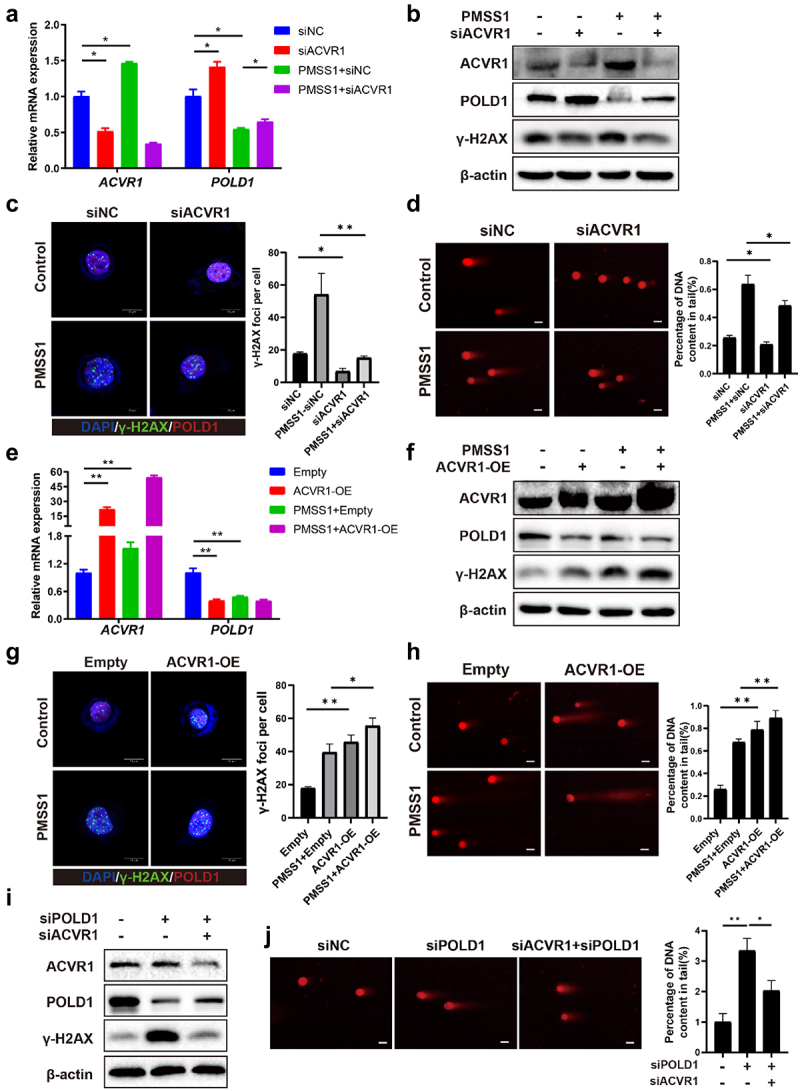
*H. pylori*infection promoted POLD1-mediated DSBs accumulation through ACVR1 activation. (a to d) AGS cells were transfected with ACVR1 knockdown siRNA and then co-cultured with *H. pylori*. qRT-pcr analysis showing the expression of POLD1 (a). Western blot analysis showing the expression levels of POLD1 and γ-H2AX (b). Immunofluorescence staining and quantification data for γ-H2AX foci formation and POLD1 expression. (Scale bar:10 um) (c). Representative images and quantification data of neutral comet assay. (Scale bar:10 µm) (d). (e to h) AGS cells were transfected with ACVR1 overexpression plasmids and then co-cultured with *H. pylori*. qRT-pcr analysis showing the expression of POLD1 (e). Western blot showing the expression levels of POLD1 and γ-H2AX. (f) Immunofluorescence staining and quantification data for γ-H2AX foci formation and POLD1 expression (Scale bar:10 µm) (g). Representative images and quantification data of neutral comet assay (Scale bar:10 µm) (h). (i and j) AGS cells were transfected with both POLD1 and ACVR1 knockdown siRNA. Western blotting was used to assess the expression levels of POLD1 and γ-H2AX (I). Representative micrographs and quantification data of neutral comet assay. (Scale bar:10 µm) (j). Data are shown as means ± SD. *P* values were calculated using Student’s t test; *, *P* < 0.05; **, *P* < 0.01; ***P < 0.001. Independent experiments were performed at least two times. The data presented are representative results.

Building upon these findings, we sought to explore whether *H. pylori* infection facilitated POLD1-mediated DSBs in an ACVR1-dependent manner. Our data demonstrated that knockdown of ACVR1 by siRNA caused a significant increase in both mRNA and protein levels of POLD1 following infection with *H. pylori* in AGS cells (Figure 4(a,b)). To elucidate the involvement of AVCR1 in DNA damage induced by *H. pylori*, we performed immunofluorescence analyses to detect γ-H2AX foci. The knockdown of endogenous ACVR1 significantly inhibited the formation of *H. pylori*-induced γ-H2AX nucleus foci (Figure 4(c)). Furthermore, silencing ACVR1 with siRNA resulted in a reduction in the comet tail length following infection with *H. pylori* (Figure 4(d)). To validate the impact of ACVR1 on POLD1 and DSBs, we transiently overexpressed ACVR1 in gastric epithelial AGS cells. As expected, the inhibition of POLD1 expression by *H. pylori* infection was enhanced upon ACVR1 overexpression (Figure 4(e)). Transient overexpression of ACVR1 also significantly enhanced the γ-H2AX levels and displayed elongated comet tails upon *H. pylori* infection (Figure 4, (f-h)). To validate the interplay between ACVR1 and POLD1 in regulating DNA damage, a rescue assay was performed. Silencing POLD1 promoted γ-H2AX expression and comet tail length, effects that were counteracted by knockdown of ACVR1 (Figure 4(i,j)).

The presence of ROS is a common factor leading to DSBs induced by *H. pylori* infection. To explore the association between ACVR1 and ROS, we assessed ROS generation and the activation status of key DNA damage response (DDR) molecules ATM and Chk2 in the specified cells. While *H. pylori* infection notably enhanced the activation of ATM and Chk2, silencing ACVR1 expression had no impact on their activation (Figure S4, A). Furthermore, alterations in ACVR1 levels did not affect ROS production (Figure S4, B).

In summary, these results indicate that *H. pylori* infection facilitates the accumulation of POLD1-mediated DSBs through the activation of ACVR1expression, independent of ROS occurrence.

### POLD1 expression is induced via direct binding of IRF3 on the POLD1 promoter

**Figure 5.**
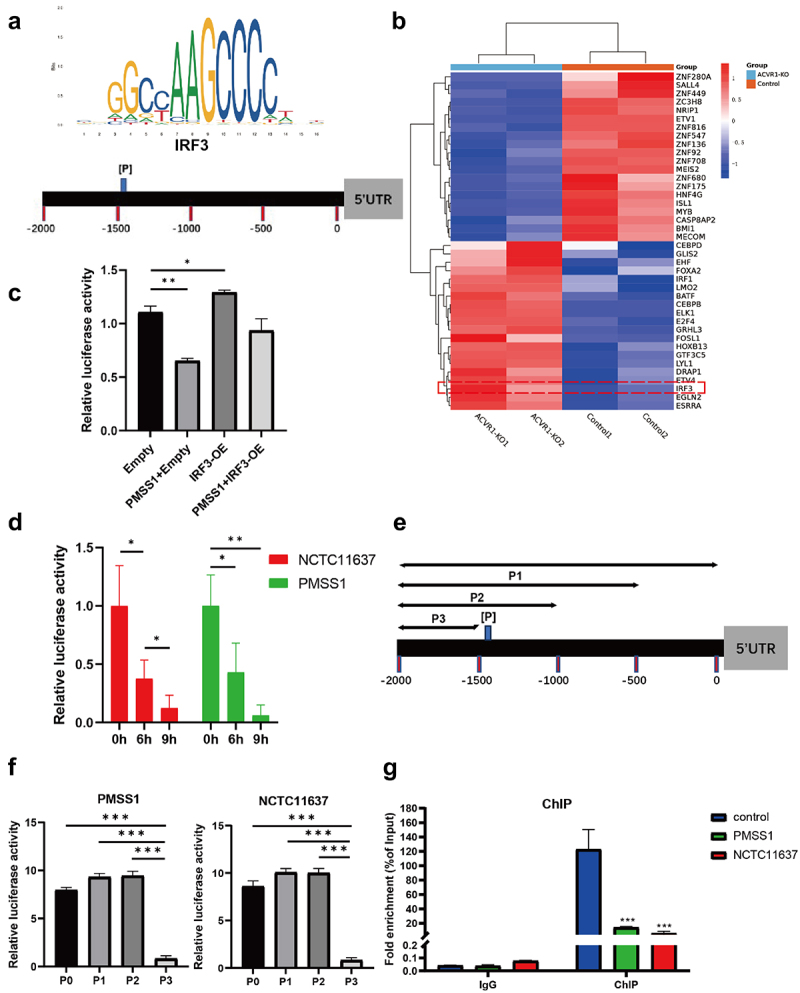
The POLD1 expression is induced through the direct binding of IRF3 to the POLD1 promoter. (a) The schematic diagram showing the locations of the putative IRF3 binding regions on the POLD1 promoter. (b) Heatmap showing the differentially expressed transcription factors. The dashed red square highlights the transcription factor IRF3. (c) AGS cells were transfected with IRF3 overexpression (OE) plasmid and then co-cultured with *H. pylori* PMSS1 strain. POLD1 luciferase promoter-reporter assay showing the luciferase activity. (d) AGS cells were infected with *H. pylori* PMSS1 and NCTC11637 strains, respectively, with different incubation time (0 h, 6 h, 9 h). POLD1 luciferase promoter-reporter assay indicating the luciferase activity. (e) POLD1 promoter plasmids containing different fragments were designed as shown in the schematic form. (f) Luciferase analysis showing the luciferase activity in AGS cells after transfection with different POLD1 promoter plasmids. (g) ChIP assay showing the amplification of DNA fragments quantified by qRT-pcr. Data are shown as means ± SD. *P* values were calculated using Student’s t test; *, *P* < 0.05; **, *P* < 0.01; ***P < 0.001.

Next, we explored the molecular mechanism of underlying ACVR1-mediated inhibition of POLD1 in response to *H. pylori* infection. Interestingly, immunofluorescence analyses elucidated that ACVR1 is localized on the cell membrane while POLD1 is present in the nucleus (Figure S3, K). Considering ACVR1‘s influence on modulating POLD1 at the mRNA and protein levels, we postulated the potential involvement of specific transcription factors in mediating ACVR1‘s impact on POLD1 expression regulation. To delve deeper into this aspect, computational analysis of the POLD1 promoter was conducted utilizing two established databases: JASPAR (http://jaspar.genereg,net) and the Animal Transcription Factor Data Base (Animal TFDB, version 3.0, http://bioinfo,life.hust.edu.cn/Animal TFDB/). This bioinformatics scrutiny revealed the presence of a putative IRF3 binding site within the regulatory region of the POLD1 gene promoter (Figure 5(a) identified as P site).

Furthermore, the analysis of ACVR1 transcriptomic data revealed a significant upregulation of IRF3 expression following ACVR1 knockout (Figure 5(b)). Utilizing a POLD1 luciferase reporter constructed harboring IRF3 binding sites, we observed a decrease in luciferase reporter activity in AGS cells upon *H. pylori* infection. Conversely, the overexpression of IRF3 enhanced luciferase reporter activity (Figure 5(c)). Notably, we also found that *H. pylori* infection suppressed POLD1 luciferase activity across various different time points (Figure 5d). Consistently, similar results were obtained in HFE145 cells (Figure S4, C and D). To identify the IRF3 binding, we generated POLD1 promoter sequences of varying lengths (Figure 5e) and transfected them into AGS and HFE145 cells. Remarkably, the P3 segment, which lacked the binding P sites, exhibited a notable decrease in luciferase activity (Figure 5(f) Figure S4, E and F). To validate the direct binding of IRF3 to the POLD1 promoter region, we designed primers covering the specific-binding sites for chromatin immunoprecipitation (ChIP) analysis. Subsequent ChIP analysis post *H. pylori* infection revealed positive amplification of the primers, affirming the binding of IRF3 to the POLD1 promoter (Figure 5g). Collectively, these results indicate that *H. pylori* repressed POLD1 expression by facilitating IRF3 binding to the POLD1 gene promoter, possibly through binding sites in the P site.

### H. Pylori *infection inhibited IRF3/POLD1 axis and triggered DNA damage through ACVR1*

**Figure 6.**
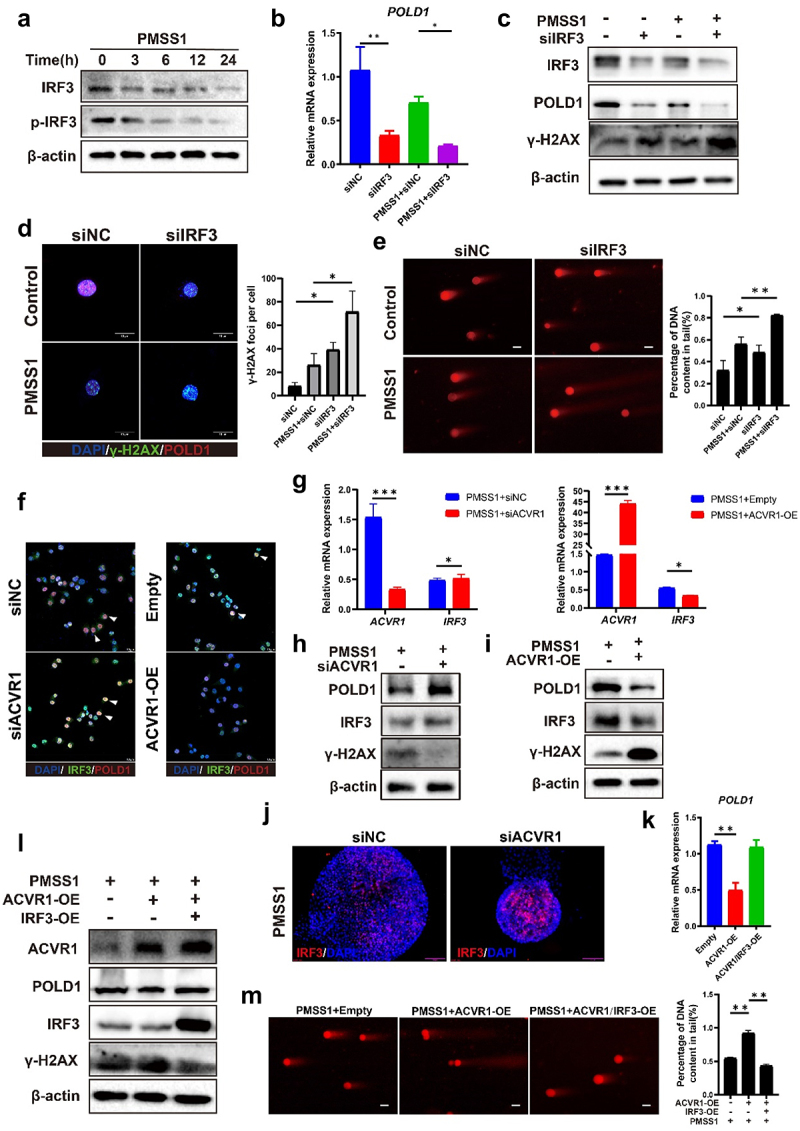
*H. pylori*infection inhibits IRF3 transcriptional activation of POLD1 and promotes DSBs. (a) Western blot analysis of the expression levels of IRF3 and p-IRF3 in AGS cells following *H. pylori*PMSS1 infection for different incubation time (0 h, 3 h, 6 h, 12 h, 24 h). (b–e) AGS cells were transfected with IRF3 knockdown siRNA and then co-cultured with *H. pylori*. qRT-pcr analysis showed the expression level of POLD1 (b). Western blot showing the expression levels of IRF3, POLD1 and γ-H2AX (c). Immunofluorescence staining and quantification data for γ-H2AX foci formation and POLD1 expression (Scale bar:10 µm) (d). Representative images and quantification data of comet assay (Scale bar:10 µm) (e). (f–j) AGS cells and gastric organoids were transfected with ACVR1 siRNA and overexpression plasmids, respectively. Immunofluorescence staining showing the subcellular localization of IRF3 and POLD1. (Scale bar:10 µm) (f). qRT-pcr analysis showed the expression level of IRF3 (g). Western blot showing the expression levels of POLD1, IRF3 and γ-H2AX (h and i). Immunofluorescence staining to determine the expression of IRF3 in gastric organoid transfected with the ACVR1 siRNA. (Scale bar, 50 µm) (j). (k to m) qRT-pcr showing the POLD1 expression in the AGS cells transfected with both ACVR1 and IRF3 overexpression plasmids (k). Western blot showing the expression levels of POLD1, IRF3 and γ-H2AX (l). The comet assay micrographs and quantification data (Scale bar:10 µm) (m). Data are representative of at least three independent experiments; ~1000 cells per condition are show in (d); ~100 cells per condition are shown in (e and m). Data are shown as means ± SD. *p* values were calculated using Student’s t test; *, *P* < 0.05; **, *P* < 0.01; ***P < 0.001.

To elucidate the association between IRF3 and *H. pylori*, we first investigated the impact of *H. pylori* infection on IRF3 activity. Infection with *H. pylori* PMSS1 strain significantly decreased the levels of total and phosphorylated IRF3 proteins (Figure 6(a)), concomitant with a suppression in mRNA expression of *IRF3* (Figure S5, A). Similar results were also observed in gastric organoids (Figure S5, B). Subsequently, to probe the correlation between IRF3 and POLD1 in the context of *H. pylori* infection, we utilized IRF3 knockdown siRNA and overexpression plasmids in AGS cells. Knockdown of endogenous IRF3 expression within AGS cells yielded a significant decrease in both POLD1 mRNA and protein levels (Figure S5, C and D), whereas overexpression of IRF3 led to an elevation in POLD1expression (Figure S5, E and F), compared to the control groups.

Furthermore, the downregulation of POLD1 expression induced by *H. pylori* infection was magnified by the knockdown of IRF3 (Figure 6(b,c)). Conversely, IRF3 overexpression partially mitigated the suppression caused by *H. pylori* infection (Figure S5, G and H). As POLD1 is one of the key enzymes in DNA damage repair, we sought to investigate whether *H. pylori* infection induced POLD1-mediated DSBs by inhibiting IRF3. Immunofluorescence staining showed that IRF3 silencing via siRNA significantly increased the nuclear foci of γ-H2AX following *H. pylori* infection (Figure 6(d)). Subsequent comet assay confirmed the exacerbation of DNA damage in cell with IRF3 knockdown (Figure 6E). Consistent with these observations, the overexpression of IRF3 inhibited the formation of γ-H2AX and comet tails induced by *H. pylori* (Figure S5, I and J).

Next, we investigated the dependency of *H. pylori*-induced inhibition of POLD1 and IRF3 on ACVR1. ACVR1 knockdown promoted the cellular colocalization of IRF3 and POLD1, while ACVR1 overexpression counteracted this effect (Figure 6(f) Figure S5, K). Furthermore, our observations indicated that *H. pylori* infection could diminish the nuclear localization of both IRF3 and POLD1 (Figure S5, L). To ascertain the casual relationship between ACVR1 and IRF3, we performed ACVR1 silencing or transient overexpression in AGS cells infected with *H. pylori*. The findings revealed that downregulation of ACVR1 enhanced IRF3 expression, whereas ACVR1 overexpression notably decreased IRF3 expression levels (Figure 6(g-i)). These results were further confirmed in gastric organoids. Specifically, upon silencing ACVR1 expression in *H. pylori*-infected organoids, a marked increase in IRF3 expression was observed (Figure 6(j)). Then, we investigated whether *H. pylori* infection promoted DNA damages through the ACVR1/IRF3 axis. Our results demonstrated that overexpression of ACVR1 facilitated *H. pylori* infection-induced γH2AX foci and comet tail formation, which were strikingly reduced after transfection with an *IRF3*-overexpressing plasmid (Figure 6(k-m)).

To further explore the interplay between ROS and the ACVR1/IRF3 axis, we conducted additional experiments in cells with IRF3 overexpression to evaluate ROS associated DDR signaling molecules. In line with previous observations, the heightened expression IRF3 did not significantly affect intracellular ROS levels or the DDR pathway (Figure S5, M and N). Conversely, we investigated whether ROS influences ACVR1/IRF3 dynamics. Utilizing N-acetyl-cysteine (NAC) as a common ROS inhibitor in *H. pylori*-infected cells, no significant alterations were noted in the expression of ACVR1, POLD1, and IRF3 (Figure S5, O). Furthermore, the restoration of ROS production, inhibited by NAC, was not achieved through ACVR1 overexpression (Figure S5, P).

Moreover, considering the established role of ACVR1 in BMP signaling pathway activation, we investigated whether the regulation of IRF3/POLD1 by ACVR1 is dependent on BMP signal transduction. Through the use of the BMP signaling agonist SB4, which stimulates the BMP signal by stabilizing intracellular p-SMAD-1/5/9 proteins, our results revealed a noteworthy reduction in IRF3 and POLD1 expression levels upon ACVR1 inhibition and subsequent BMP pathway activation (Figure S5, Q). This suggests that ACVR1‘s impact on IRF3 expression may be mediated via the BMP signaling pathway.

Collectively, our data suggest that *H. pylori* infection induces ACVR1 to suppress the IRF3/POLD1 signaling axis independently of ROS presence, culminating in DSBs.

### *Treatment with an ACVR1 inhibitor significantly ameliorated the gastric pathology induced by* H. pylori *infection in INS-GAS mice*

**Figure 7.**
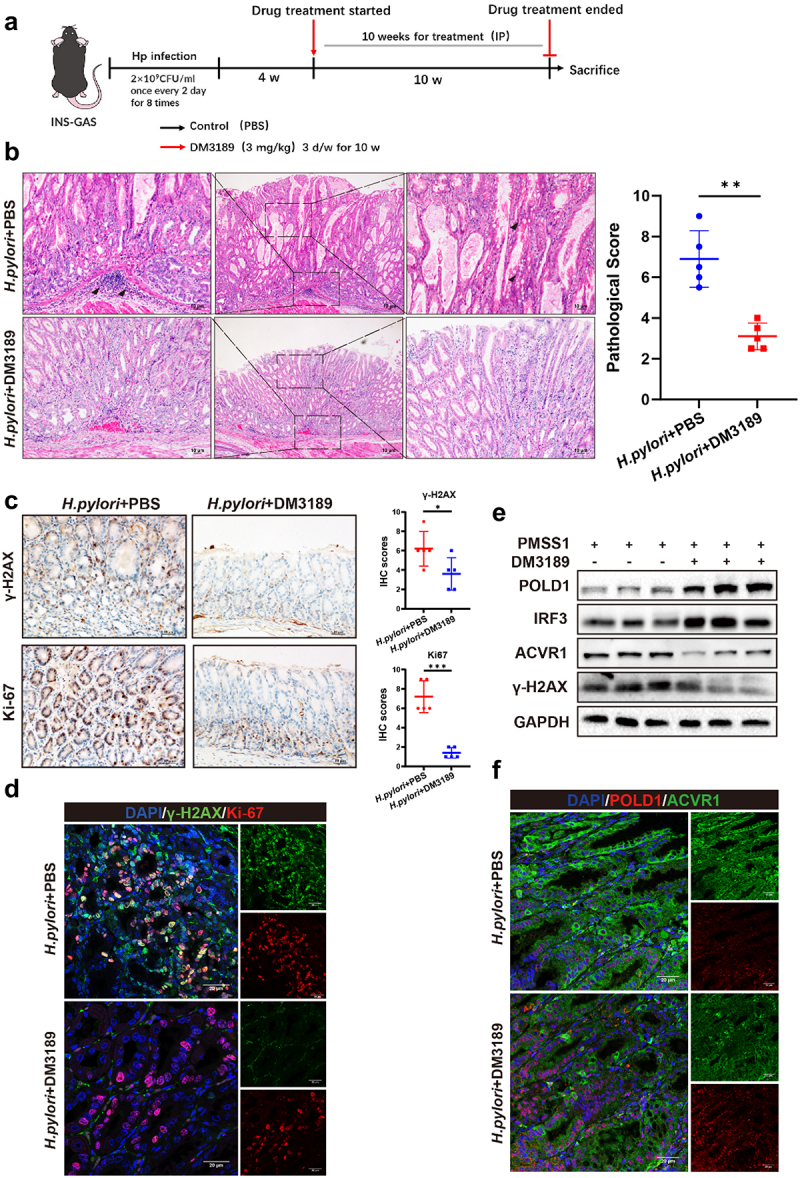
ACVR1 inhibitor ameliorated the gastric pathology induced by *H. pylori* infection. (a) Experimental design using INS-GAS mouse model. Mice were infected with *H. pylori* PMSS1 strains for 1 month, followed by intraperitoneal injection with 3 mg/kg of DM3189 for 10 weeks. (three times a week) (b) H&E staining of gastric mucosa from INS-GAS mice (Scale bar: 10 µm) (*n* = 5 mice for each group). The histopathological features of gastric mucosa for all mice were analyzed. (c) Representative immunohistochemistry staining (Scale bar:10 µm) and quantification analysis of γ-H2AX and Ki-67 in stomach tissues from the mice. (*n* = 5 for each group). (d) Immunofluorescence staining showing the localization of Ki-67 and γ-H2AX in stomach tissues from the mice (Scale bar:10 µm). (e) Western blot analysis of POLD1, IRF3, ACVR1 and γ-H2AX in stomach tissues from the infected mice after treatment with DM3189 (*n* = 3 for each group). (f) Immunofluorescence staining showing the localization of ACVR1 and POLD1 in stomach tissues from the mice (Scale bar:10 µm). Data are shown as means ± SD. *P* values were calculated using Student’s t test; *, *P* < 0.05; **, *P* < 0.01; ***P < 0.001. Independent experiments were performed at least two times. The data presented are representative results.

To evaluate the role of ACVR1 in *H. pylori* infection-induced pathology, we administered the ACVR1 inhibitor DM3189 to INS-GAS mice infected with *H. pylori* (Figure 7(a)). Silver staining and colony formation assays showed no significant difference in *H. pylori* colonization of the gastric mucosa between mice infected with *H. pylori* alone and those treated with the ACVR1 inhibitor DM3189 (Figure S6, A and B). The qRT-PCR analysis confirmed that treatment with DM3189 effectively suppressed the mRNA levels of ACVR1 (Figure S6, C). Histological assessment revealed gastric inflammation and partial glandular metaplasia in PMSS1-infected mice, while treatment with the ACVR1 inhibitor DM3189 significantly alleviated gastric pathology (Figure 7(b) Figure S6, D). Furthermore, the mRNA levels of the proinflammatory cytokines *IL-1β* and *IL-18* were decreased in stomach tissues of infected mice treated with the ACVR1 inhibitor DM3189 (Figure S6, E).

Of note, the administration of DM3189 significantly inhibited the expression of cell proliferation marker Ki67 and DNA damage marker γ-H2AX in the gastric mucosa of mice post *H. pylori* infection (Figure 7(c)). And we also observed a significant increase in p53 levels in the inhibitor group (Figure S6, G). Immunofluorescence staining confirmed that treatment with the ACVR1 inhibitor led to a reduction in the colocalization of Ki67 and γ-H2AX in gastric mucosa cells of infected mice (Figure 7(d)). Moreover, in comparison to the *H. pylori* mono-infection group, treatment with DM3189 remarkably increased the expression levels of POLD1 and IRF3, while reducing γ-H2AX in infected gastric tissues (Figure 7(e,f) Figure S6, F). Additionally, evaluation of other organ lesions in mice showed no significant adverse effects of DM3189, indicating its favorable safety profile (Figure S6, H). In conclusion, these findings suggest the promising potential of ACVR1 inhibitor in the treatment of *H. pylori* infection-related gastric mucosal lesions.

### *The clinical significance of ACVR1, POLD1, and γH2AX in human gastric tissues and their relation to* H. pylori *infection*

**Figure 8.**
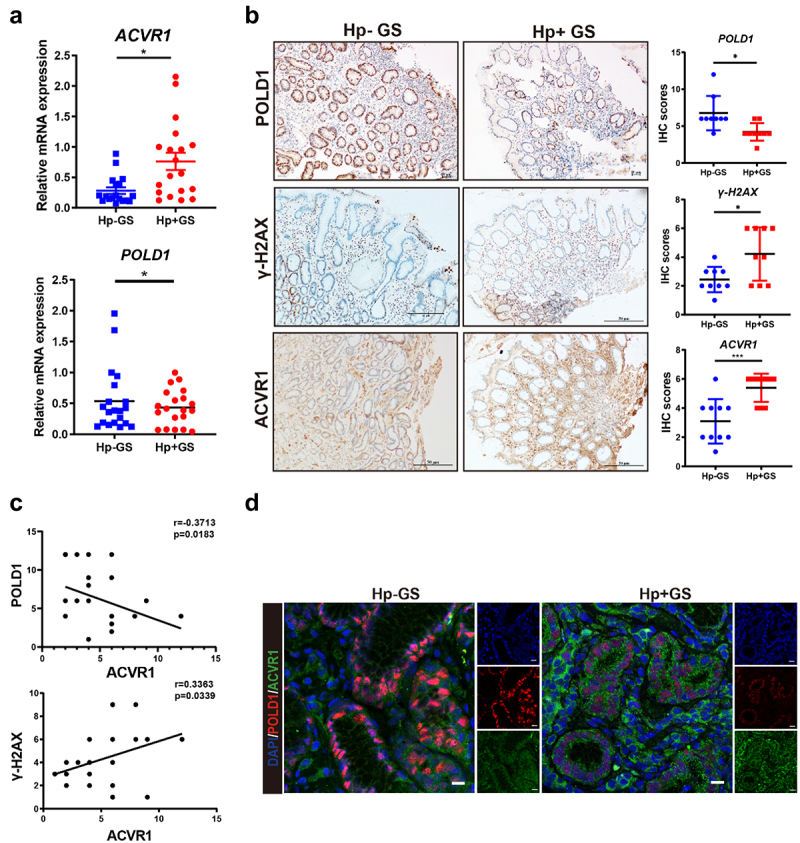
The clinical significance of ACVR1, POLD1 and γH2AX in human gastric tissues and their relationship with *H. pylori* infection. (a) qRT-pcr analysis showing the mRNA levels of ACVR1 and POLD1 in *H. pylori* infected human gastritis (*H. pylori*-gs: n = 19; *H. pylori*+GS: n = 19). (b) Representative immunohistochemical staining and quantification analysis of ACVR1, POLD1 and γ-H2AX in *H. pylori* infected human gastritis tissues. (Scale bar: 10 µm) (n = 18). (c) Spearman’s correlation between IHC staining scores of ACVR1, POLD1 and γ-H2AX in *H. pylori* infection human gastritis tissues (n = 36). (d) Immunofluorescence staining showing the localization of ACVR1 and POLD1 in stomach tissues from human tissues (body) (Scale bar: 10 µm). Data are shown as means ± SD. *p* values were calculated using Student’s t test; *, *P* < 0.05; **, *P* < 0.01; ***P < 0.001.

In continuation of our *in vitro* and in *vivo* investigations, we proceeded to assess the clinical significance of ACVR1, POLD1, and γH2AX in human gastric tissues. The qRT-PCR analysis showed that the mRNA levels of ACVR1 was higher in *H. pylori* positive gastritis tissues, compared with *H. pylori* negative tissues, while POLD1 exhibited the opposite pattern (Figure 8(a)). Similarly, we observed strong immunostaining of ACVR1 and γ-H2AX in gastritis tissues with *H. pylori* infection. In a marked contrast to the uninfected tissues, gastritis tissues infected with *H. pylori* exhibited weaker immunostaining of POLD1 (Figure 8(b)). Spearman’s correlation analysis demonstrated a significant inverse correlation between ACVR1 and POLD1 expression (Figure 8(c), *R* = 0.3713, *p* = 0.0183), along with a significant direct correlation between ACVR1 and γ-H2AX expression (Figure 8(c), *R* = 0.3363, *p* = 0.0339). Immunofluorescence staining further highlighted an appreciable elevation in ACVR1 expression coupled with a meaningful decrease in POLD1 expression in *H. pylori*-positive patients suffering from gastric inflammation (Figure 8(d)).

Collectively, these observations on the expression levels of ACVR1, POLD1 andγ-H2AX in gastric tissues, both with or without *H. pylori* infection, lend clinical support to the association between DNA damage and ACVR1 activation in the process of *H. pylori*-induced gastric carcinogenesis.

## Discussion

Our previous study highlighted a potential involvement of ACVR1 in gastric cancer development triggered by *H. pylori*.^28^ The current study sought to elucidate the impact of ACVR1 on *H. pylori*-induced gastric carcinogenesis, focusing on the underlying mechanism of elevated ACVR1 expression. Despite documented upregulation of ACVR1 in various tumors, its specific role in the context of *H. pylori* infection and gastric carcinogenesis remains incompletely understood.^37–39^ Employing in *vitro* cells, gastric organoids, mouse models and human tissue specimens, we found that ACVR1 inhibits the phosphorylation of IRF3, leading to transcriptional suppression of POLD1, which plays a crucial role in HR repair, influencing the occurrence of DSB. Furthermore, our findings suggest that inhibition of ACVR1 may hold therapeutic promise in alleviating *H. pylori*-induced gastric mucosal lesions.

ACVR1, a constituent of the transforming growth factor-beta (TGF-β) family, has been recognized for its involvement in tumor initiation and progression across various cancer types.^40–43^ Wang and colleagues found that stress-induced phosphoprotein 1 (STIP1), a protein adaptor and modulator of heat shock proteins HSP70 and HSP90, was secreted by ovarian cancer cells and detected in the blood of ovarian cancer patients.^44^ STIP1 was observed to directly interact with ACVR1, activating it and enhancing SMAD1/5 phosphorylation downstream of ACVR1. This activation signal was linked to the proliferation of ovarian cancer cells through this ACVR1-SMAD signaling pathway.^45^ In prostate cancer, ACVR1 was demonstrated to phosphorylate endothelial factors, facilitating prostate cancer cell metastasis.^46^ Moreover, ACVR1 has been implicated in promoting carcinogenesis in endometrial cancer, multiple myeloma, leukemia, and various other malignancies.^47–49^ However, its involvement in tumors associated with chronic inflammatory infections remains unexplored. *H. pylori* is classified as a Group I carcinogen for gastric cancer and represents the primary etiological factor in most cases of intestinal-type gastric cancers.^50,51^

In our previous studies, we unveiled a novel insight demonstrating that *H. pylori* infection activates ACVR1 independently of CagA, thereby promoting the transcriptional activity of CDX2 and initiating intestinal metaplasia.^52^ Building on these findings, the present study embarks on a comprehensive investigation into the interplay between ACVR1 and gastric cancer pathogenesis. Our investigations revealed that the activation of ACVR1 by *H. pylori* occurs independently of the virulence factor VacA. It is plausible that *H. pylori* might activate ACVR1 through alternative virulence proteins, such as HtrA and Outer Membrane Vesicles (OMVs). This suggests a promising avenue for further comprehensive exploration in our forthcoming research endeavors.^53^ The INS-GAS mice are a transgenic mouse model, characterized with gastric somatostatin overexpression, has emerged as a valuable tool for studying gastric cancer development in response to *H. pylori* infection.^54–58^ Following 4 months of *H. pylori* infection, INS-GAS mice exhibited notable inflammation infiltration and partial glandular metaplasia in the stomach, concomitant with the extensive activation of ACVR1, particularly pronounced at the site of *H. pylori* colonization. This pivotal observation marks the initial identification of the association between ACVR1 and gastric cancer onset triggered by *H. pylori* infection.

DNA double-strand breaks (DSBs) represent a prevalent and severe form of DNA damage associated with tumorigenesis, as evidenced by numerous studies.^11,59^ γ-H2AX, a hallmark protein indicative of DSB formation, is commonly utilized in DNA damage research.^19,60^ Prior research endeavors, including our own preliminary investigations, have elucidated the ability of *H. pylori* to induce DSBs through diverse mechanisms.^10,17,20,61^ *POLD1*, a crucial gene encoding the catalytic subunit of DNA polymerase delta, plays an indispensable role in various DNA repair pathways that safeguard chromosomal stability and prevent genetic variations.^13^ Upon DSB occurrence, POLD1 is recruited by ATR to the sites of DNA damage, facilitating lagging strand synthesis and subsequent restoration of the DNA double helix structure. Frequent mutations and inactivation of POLD1 are commonly observed in tumorigenesis. Studies by Church and Castillo have linked mutations in POLD1 to a poorer prognosis in patients with endometrial cancer.^62,63^ Additionally, POLD1 inactivation can increase the risk of gastrointestinal tumor development.^64^ Mutations in POLD1 have been identified as playing a significant role in the initiation and progression of ovarian cancer and endometrial cancer.^52,65^ Over all, substantial scientific literature has established that mutations or deficiencies in POLD1 contribute to genomic instability and the pathogenesis of various diseases.^66–68^ Nevertheless, the association between POLD1 and tumors triggered by chronic infections has not been definitively elucidated.

Therefore, through transcriptomic analysis in our study, we unveil a novel discovery that *H. pylori* infection exerts a suppressive effect on POLD1 and disrupts the homologous recombination (HR) repair pathway via ACVR1 activation, marking the first documentation of such an interaction. This novel finding not only enhances our comprehension of the mechanisms underlying DSB generation during *H. pylori* infection but also provides novel evidence for the involvement of *H. pylori* in gastric cancer development. Remarkably, the results from Kontizas’s investigation corroborate our findings by highlighting *H. pylori*-induced suppression of POLD1.^30^ Furthermore, our previous investigations delineated that *H. pylori* infection hampers RAD51 ubiquitination, consequently impeding HR repair. However, in this study, we demonstrate that ACVR1 activation does not suppress Rad51 expression. Hence, we propose that the activation of ACVR1 by *H. pylori* represents a novel tactic employed by the bacterium to circumvent DNA repair processes, shedding light on a previously unrecognized mechanism underpinning microbial manipulation of host cellular responses.

Reactive oxygen species (ROS) are commonly associated with the occurrence of double-strand break (DSB).^69^ Previous studies have indicated that *H. pylori* infection can stimulate the production of reactive oxygen species (ROS) through various mechanisms, contributing to DSBs formation.^70,71^ In our current study, we sought to explore the relationship between the ACVR1-IRF3-POLD1 axis and ROS generation. By modulating the expression of ACVR1 and IRF3 separately and monitoring ROS levels, we discovered that neither sliencing ACVR1 nor overexpressing IRF3 affected intracellular ROS production. The activation of the key DDR pathway effector molecule ATM, Chk2 related to ROS was also unaffected. Moreover, we investigated the impact of ROS on ACVR1-IRF3-POLD1 pathway. Upon treatment with the ROS inhibitor N-acetyl-cysteine (NAC), there were no significant alterations in the expression of IRF3 and POLD1. These results indicate that the activation of the ACVR1-IRF3-POLD1 pathway is independent of intracellular ROS generation.

IRF3, belonging to the interferon regulatory factor (IRF) family, is recognized as a pivotal early orchestrator of virus sensing.^72^ When cells are subjected to external stimuli such as viral infection, IRF3 typically undergoes activation as part of the cellular defense response.^73^ Our investigations revealed that *H. pylori* infection suppressed both IRF3 mRNA and protein levels, aligning with previous study indicating that *H. pylori* infection suppresses the transcriptional activity of IRF3, thereby facilitating immune evasion.^74^ Interestingly, conflicting reports indicate that *H. pylori* infection can activate the cGAS pathway by upregulating IRF3 expression.^75^ The disparities might be attributed to variations in IRF3 expression levels influenced by the duration of the infection. It is conceivable that during early-stage *H. pylori* infection, activation of the organism’s stress response triggers IRF3 activation, whereas prolonged infection stimuli may downregulate IRF3 expression, thereby fostering the occurrence of DSB. Moreover, we demonstrated direct binding of IRF3 to the POLD1 promoter, initiating transcriptional activation of POLD1. Intriguingly, *H. pylori* infection disrupts the intricate interplay by activating ACVR1. This study introduces, for the first time, the involvement of the ACVR1-IRF3-POLD1 signaling axis in mediating the inhibition of DNA repair processes during *H. pylori* infection, thereby opening up new avenues for future investigations into the mechanisms implicated in *H. pylori*-related gastric cancer development. It is essential to acknowledge the limitations of our study, as we did not extensively explore how ACVR1 inhibits IRF3 expression, setting the stage for further investigation as part of our ongoing research efforts.

The ACVR1 inhibitor, DM3189, recognized as a selective inhibitor of BMP signaling, exhibits notable efficacy in suppressing the transcriptional activity of ACVR1.^76^ Previous studies have reported that DM3189 effectively targets the BMP type I receptor, leading to a reduction in ectopic ossification. Furthermore, this compound has demonstrated efficacy in mitigating tumor growth and inhibiting bone formation in PCa-11b tumor-bearing mice.^77^ Another study found that administration of DM3189 hinders tumor initiation and immune evasion in tumor-initiating cells.^78^ Additionally, DM3189 displays promise in mitigating inflammation and impeding the progression of lung adenocarcinoma, exhibiting a favorable safety profile.^79,80^

In this study, we assessed the therapeutic potential of DM3189 in ameliorating gastric mucosal lesions induced by *H. pylori* infection in INS-GAS mice. After a 10-week treatment regimen, the untreated mice displayed prominent inflammatory infiltration, disrupted glandular arrangement, cystic dilation of some glands, and intestinal metaplasia in the gastric mucosa. In contrast, the mice receiving DM3189 treatment exhibited marked improvement in gastric mucosal lesions, characterized by the absence of intestinal metaplasia or glandular disruption. Additionally, immunohistochemistry analysis revealed that inhibitor treatment resulted in reduced expression of Ki-67 and γ-H2AX, consequently mitigating cell damage and abnormal proliferation induced by *H. pylori*. The elevated levels of p53 indicated that the inhibitor reversed *H. pylori*-induced inhibition of DNA repair processes.^81^ This enhanced repair activity played a role in reducing lesion formation. Hematoxylin and eosin (HE) staining of other organs indicated the excellent safety profile of the inhibitor.

Notably, *H. pylori* infection is a well-recognized trigger for gastric mucosal lesions, and eradication of *H. pylori* has been linked to substantial decrease in gastric cancer incidence.^8,82^ However, a subset of *H. pylori*-positive individuals still progresses to gastric cancer despite undergoing eradication therapy.^83^ We propose that *H. pylori* may serve as a “promoter,” whereby bacterial eradication fails to reverse the already initiated cascading reactions driving gastric cancer development. Hence, identifying suitable drugs to interrupt this process is of paramount importance. Our study presents the first evidence that sustained administration of the ACVR1 inhibitor DM3189 improves *H. pylori*-induced gastric mucosal lesions, offering a promising therapeutic strategy for managing post-eradication gastric mucosal lesions. However, considering the relatively short duration of *H. pylori* infection and the absence of significant precancerous alterations in INS-GAS mice, further investigations involving an extended infection period are warranted to elucidate the pronounced therapeutic effects of DM3189.

By elucidating the intricate interplay among ACVR1, IRF3, POLD1, and DNA repair mechanisms, this study provides valuable insights into the pathogenesis of *H. pylori*-associated gastric carcinogenesis and underscores the potential therapeutic implications of targeting the ACVR1 pathway to mitigate the adverse effects of *H. pylori*-induced gastric mucosal damage. In conclusion, our findings highlight that *H. pylori* infection influences the induction of double-strand breaks (DSBs) through the inhibitory action of ACVR1 on POLD1. We elucidate the direct binding and regulatory function of IRF3 in modulating POLD1 expression. Furthermore, we demonstrate that ACVR1 regulates POLD1 expression by suppressing IRF3 activity. This novel *H. pylori*-ACVR1-IRF3-POLD1 axis establishes a connection between infection, genome instability and the onset of gastric tumorigenesis. Utilizing ACVR1 inhibitors may represent a new promising therapeutic strategy to alleviate gastric cancer lesions induced by *H. pylori* infection.

## Supplementary Material

Supplemental Material

## Data Availability

The authors confirm that the data supporting the findings of this study are available within the article and its supplementary materials. And the data are also available upon request by contact with the corresponding author.
