# Supplementary material for: Helicobacter pylori infection induces DNA double-strand breaks through the ACVR1/IRF3/POLD1 signaling axis to drive gastric tumorigenesis
Source: Gut Microbes. 2025 Feb 9;17(1):2463581. doi: 10.1080/19490976.2025.2463581 (PMC11812335; doi:10.1080/19490976.2025.2463581)
Supplement: Supplemental Material [file KGMI_A_2463581_SM8674.zip › Supplementary Material.docx]

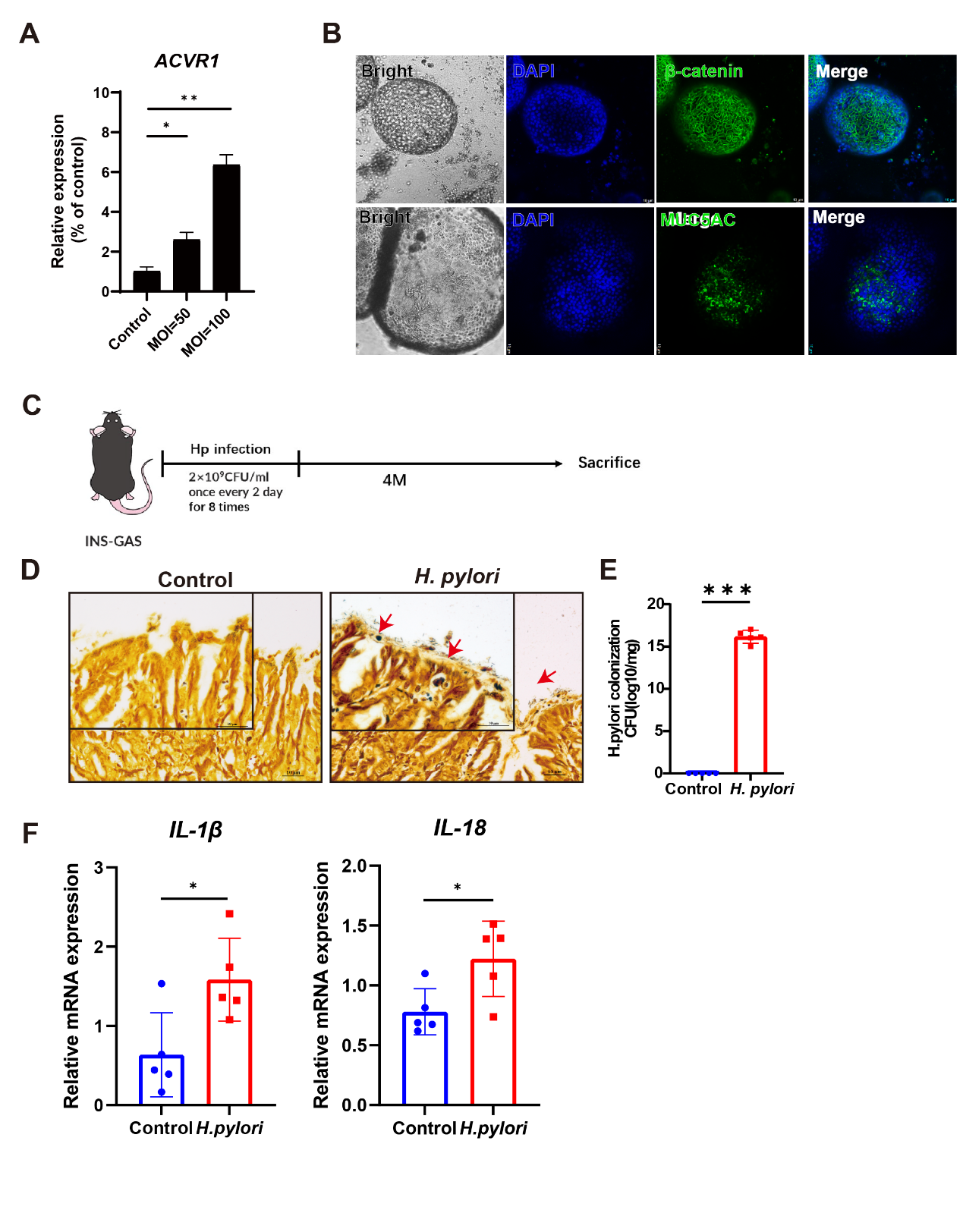


**Fig. S1.** (**A**) The quantification analysis of ACVR1 expression in AGS cells following *H. pylori* PMSS1 infection with different MOI. (**B**) Immunofluorescence staining to determine the subcellular localization of β-catenin and MUC5AC in gastric organoid (Scale bar,10 µm). (**C**) Experimental design using the INS-GAS mouse model. Mice were infected with *H. pylori* PMSS1 strains for 4 months. (**D** and **E**) Silver staining and CFU (colony forming units) showing the colonization of *H. pylori* (Black arrow) in mice stomach tissues. (Scale bar,10 µm). (**G**) qRT-PCR analysis showing the expression levels of IL-18 and IL-1β in *H. pylori* infected INS-GAS mice. Data are shown as means ± SD. P values were calculated using Student’s t test; *, *P*<0.05; **, *P*<0.01; ****P*<0.001. Experiments were performed independently for at least two times. The data presented are representative results.


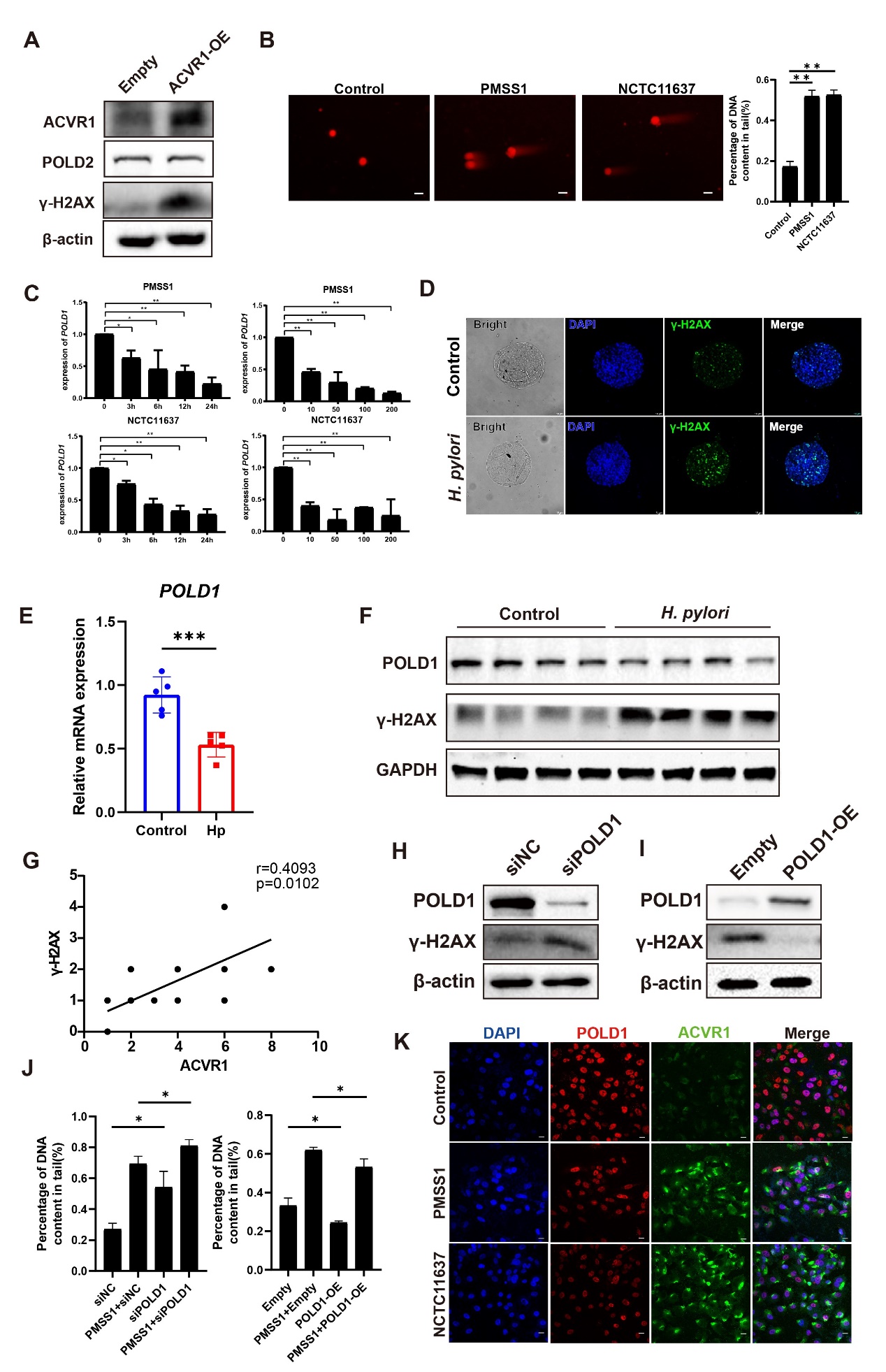


**Fig. S2.** (**A**) Western blot analysis showing the expression levels of POLD2 and γ-H2AX in AGS cells after transfected with ACVR1 knockdown siRNA. (**B**) Representative image and quantification data of comet assay from the *H. pylori* infected AGS cells (Scale bar,10 µm). (**C**) qRT-PCR analysis of the ACVR1 expression in AGS cells following infection with *H. pylori* PMSS1 and NCTC11637 strains with different infection time and MOI. (**D**) Immunofluorescence staining to determine the subcellular localization of γ-H2AX in gastric organoid infected with the *H. pylori* PMSS1 strain (Scale bar,10 µm). (**E**) qRT-PCR analysis of POLD1 expression in *H. pylori* infected mice compared with control groups. (**F**) Western blot analysis of POLD1 and γ-H2AX expression in *H. pylori* infected INS-GAS mice. (G) Spearman’s correlation of the IHC staining scores between ACVR1 and γ-H2AX. (R=0.4093, P=0.0102). (**H** and **I**) Western blot showing the expression levels of POLD1 and γ-H2AX in the AGS cells transfected with POLD1 knockdown siRNA (**H**) and overexpression plasmids (**I**). (**J**) The quantification data of comet assay from the *H. pylori* infected AGS cells post transfection with POLD1 knockdown siRNA and overexpression plasmids. (**K**) Immunofluorescence staining showing the subcellular localization of ACVR1 and POLD1 in AGS cells infected with *H. pylori* PMSS1 and NCTC11637 for 24 h, 100 MOI (Scale bar,10 µm). Data are shown as means ± SD.; ~100 cells per condition are shown in (**B** and **J**). *P* values were calculated using Student’s t test; *, *P*<0.05; **, *P*<0.01; ****P*<0.001. Independent experiments were performed at least two times. The data presented are representative results.


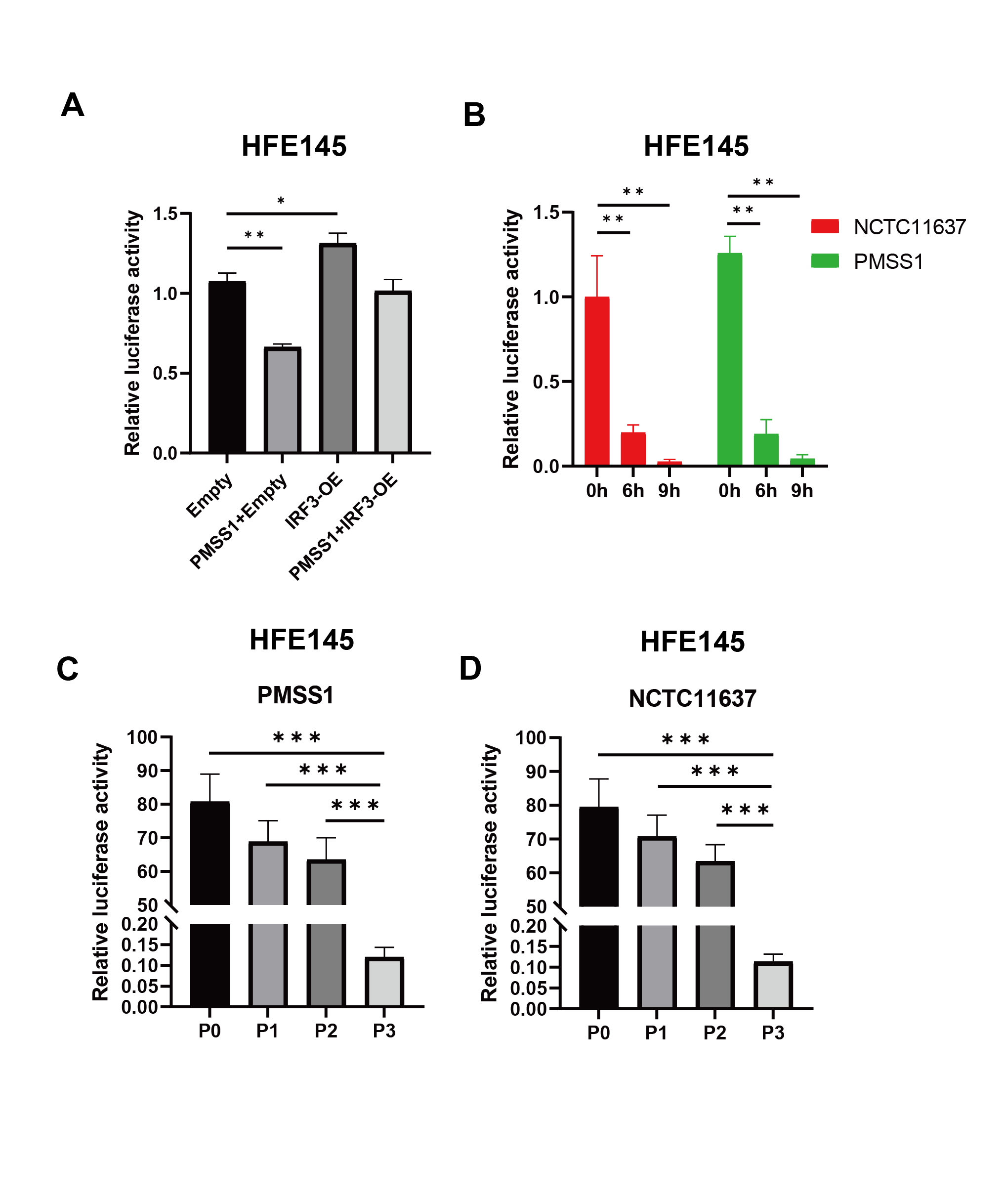


**Fig. S3.** (**A**) HFE145 cells were transfected with IRF3 overexpression plasmids and then co-cultured with *H. pylori* PMSS1 strain. POLD1 luciferase promoter-reporter assay showing the luciferase activity. (**B**) HFE145 cells were infected with *H. pylori* PMSS1 and NCTC11637 strains, respectively, for different time (0 h, 6 h, 9 h). POLD1 luciferase promoter-reporter assay displaying the luciferase activity. (**C** and **D**) Luciferase analysis showing the luciferase activity in HFE145 cell after transfection with different plasmids. Data are shown as means ± SD. *P* values were calculated using Student’s t test; *, *P*<0.05; **, *P*<0.01; ****P*<0.001.


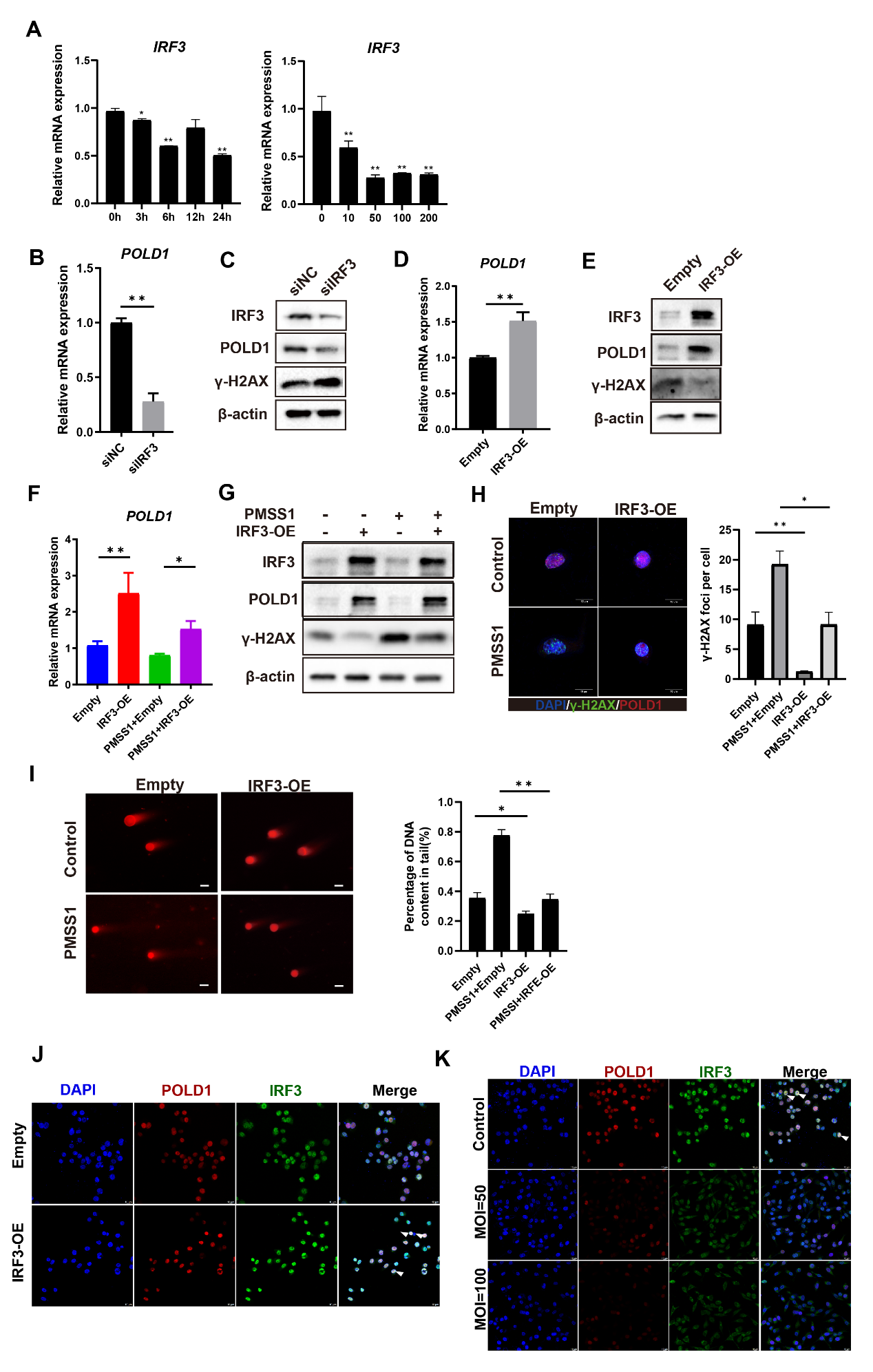


**Fig. S4**. (**A**) qRT-PCR analysis of the POLD1 expression in AGS cells following infection with *H. pylori* PMSS1 and NCTC11637 for different incubation time and with different MOIs. (**B** and **C**) AGS cells were transfected with IRF3 knockdown siRNA. qRT-PCR analysis (**B**) and western blot analysis (**C**) showing the expression of POLD1. (**D** and **E**) AGS cells were transfected with IRF3 overexpression plasmids. qRT-PCR analysis (**D**) and western blot analysis (**E**) showing the expression of POLD1. (**F** to **I**) AGS cells were transfected with IRF3 overexpression plasmids and then co-cultured with *H. pylori*. qRT-PCR analysis showing the POLD1 expression (**F**). Western blot analysis showing the expression levels of IRF3, POLD1 and γ-H2AX (**G**). Immunofluorescence images and quantification data for γ-H2AX foci formation and POLD1 expression (Scale bar:10 µm) (**H**). Representative images and quantification data of comet assay from the indicated AGS cells (Scale bar:10 µm) (**I**). (**J**) Immunofluorescence staining to determine the subcellular colocalization of IRF3 and POLD1 in the AGS cells after transfected with IRF3 overexpression plasmids (Scale bar:10 µm). (**K**) Immunofluorescence staining to determine the subcellular colocalization of IRF3 and POLD1 in the AGS cells after *H. pylori* infection with different MOI, 24 h (Scale bar:10 µm). Data are shown as means ± SD. *P* values were calculated using Student’s t test; *, *P*<0.05; **, *P*<0.01; ****P*<0.001. Independent experiments were performed at least two times. The data presented are representative results.


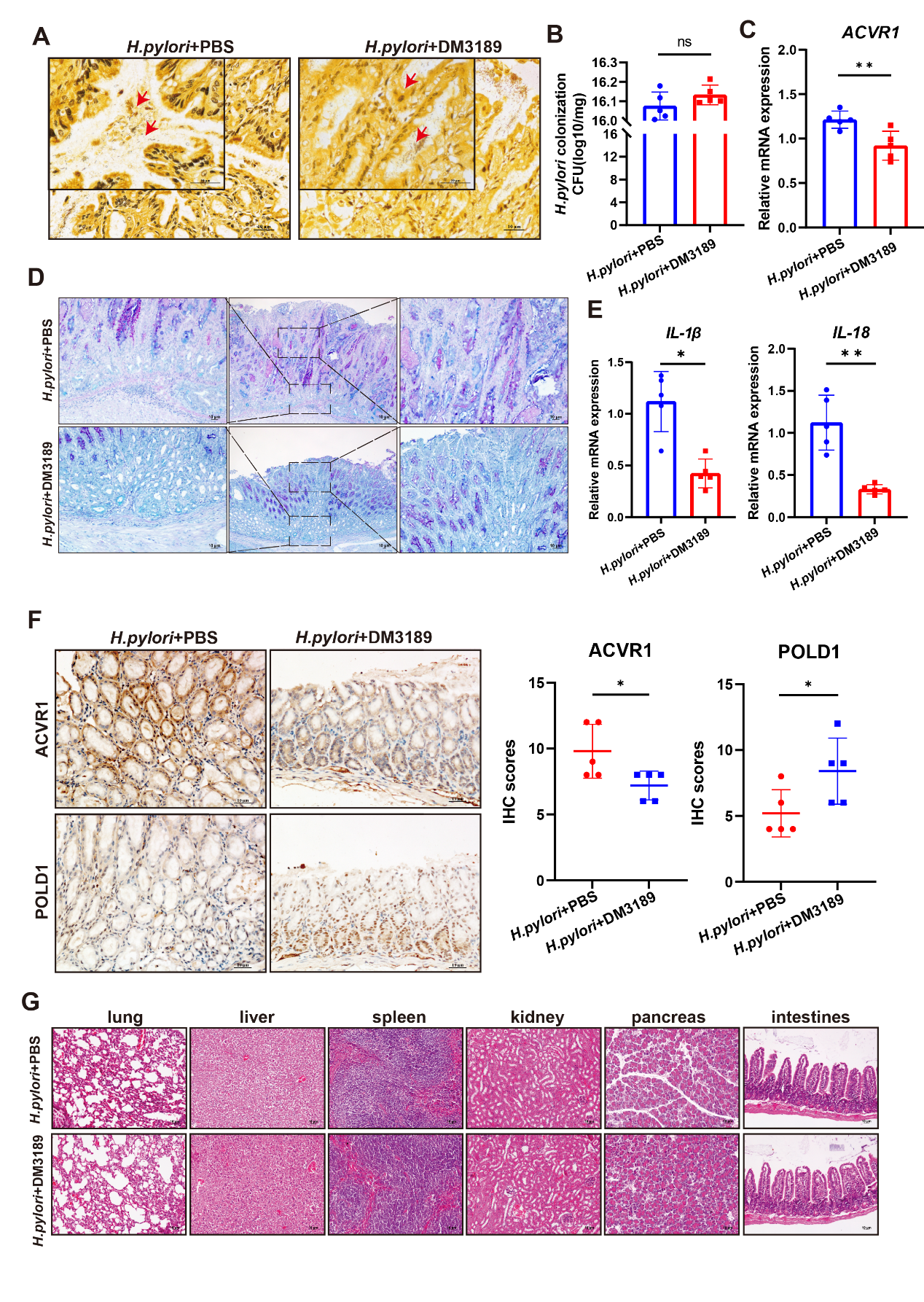


**Fig. S5.** (**A** and **B**) Silver staining and CFU (colony forming units) showing the colonization of *H. pylori* in mice stomach tissues (Scale bar:10 µm) (n=5 per group). (**C**) qRT-PCR analysis showing the expression of ACVR1 in mice with DM3189 treatment (n=5 per group). (**D**) PAS staining of gastric mucosa from INS-GAS mice (Scale bar:10 µm) (n=5 per group). (**E**) qRT-PCR analysis showing the expression levels of IL-18 and IL-β. (**F**) Representative immunohistochemistry staining and quantification analysis of ACVR1 and POLD1 in stomach tissues from the mice (Scale bar:10 µm) (n=5 per group). (**G**) H&E staining of some other selected organs from the INS-GAS mice. (Scale bar:10 µm). Data are shown as means ± SD. *P* values were calculated using Student’s t test; *, *P*<0.05; **, *P*<0.01; ****P*<0.001; ns: not significant. Independent experiments were performed at least two times. The data presented are representative results.
