# Supplementary figures and images for: Helicobacter pylori infection induces DNA double-strand breaks through the ACVR1/IRF3/POLD1 signaling axis to drive gastric tumorigenesis
Source: Gut Microbes. 2025 Feb 9;17(1):2463581. doi: 10.1080/19490976.2025.2463581 (PMC11812335; doi:10.1080/19490976.2025.2463581)

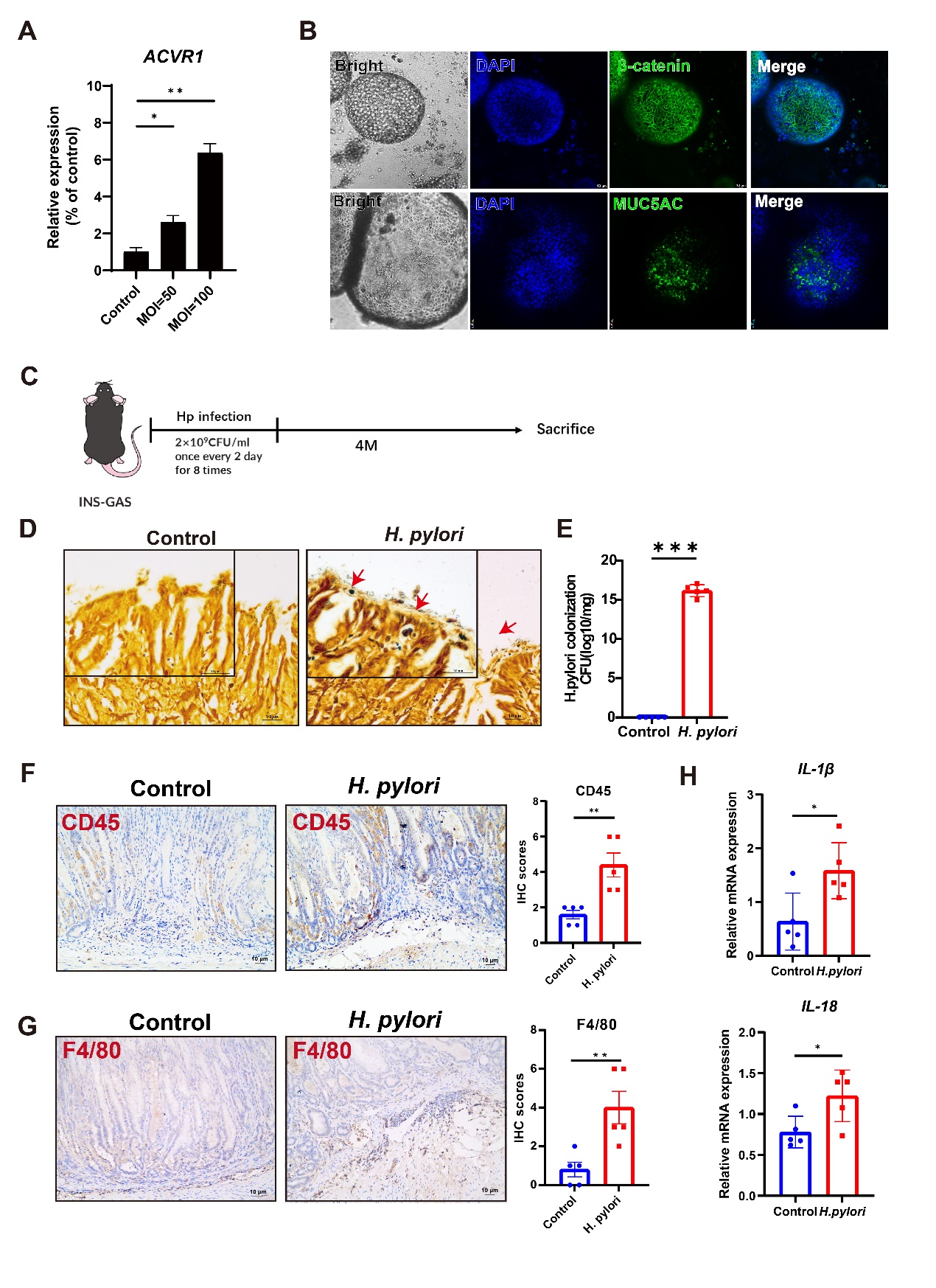


Figure s1


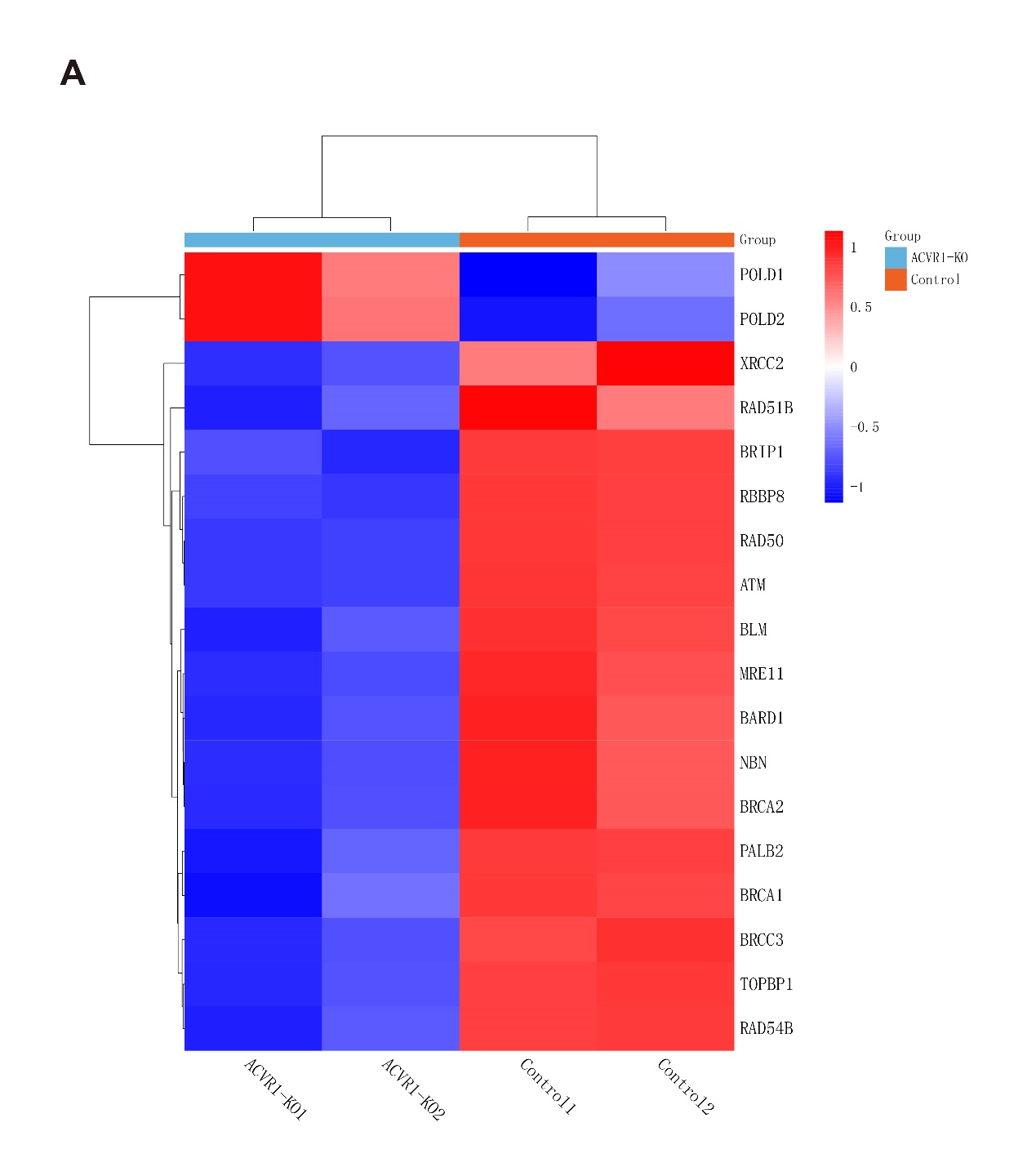


Figure S2


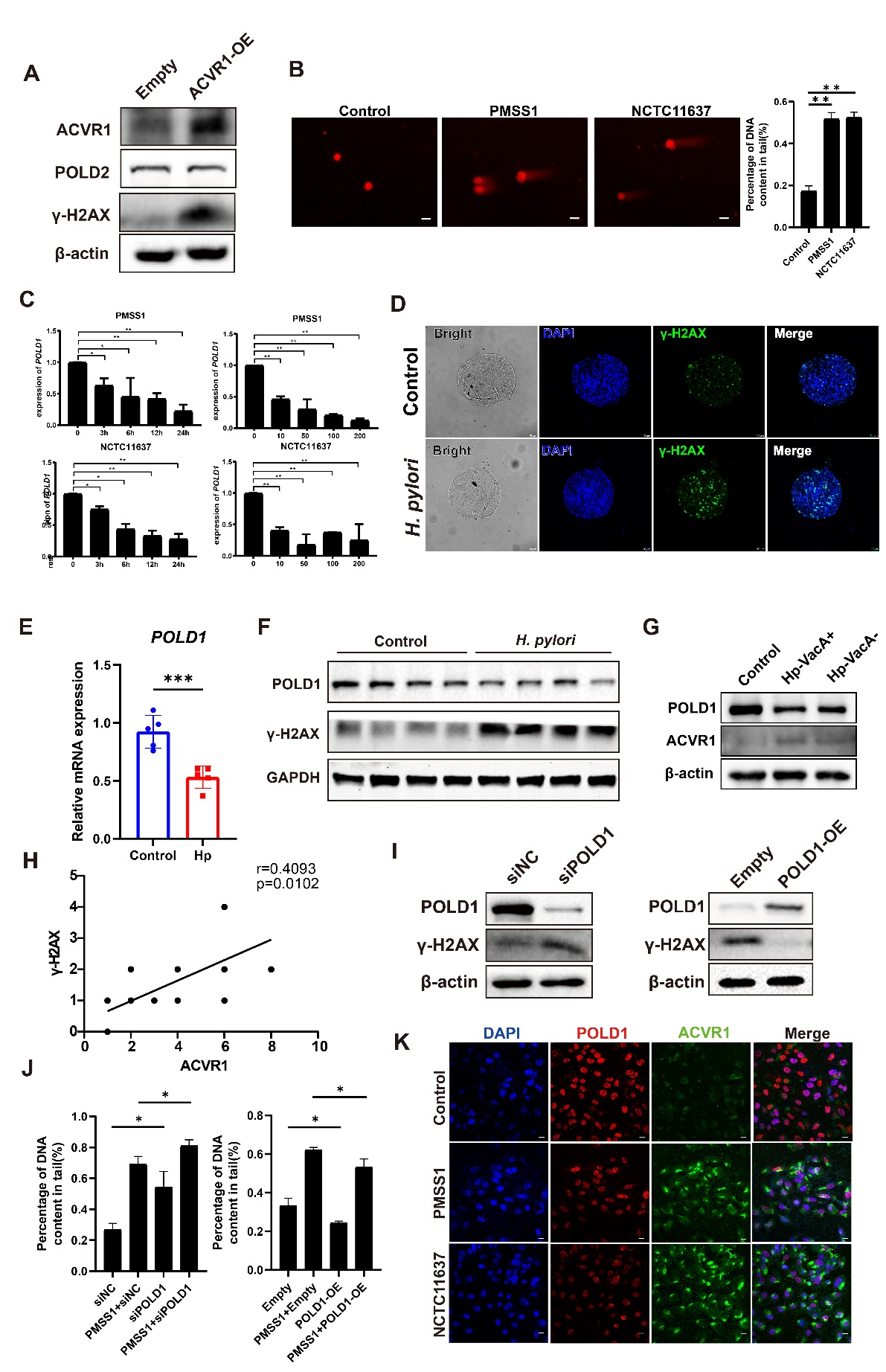


Figure S3


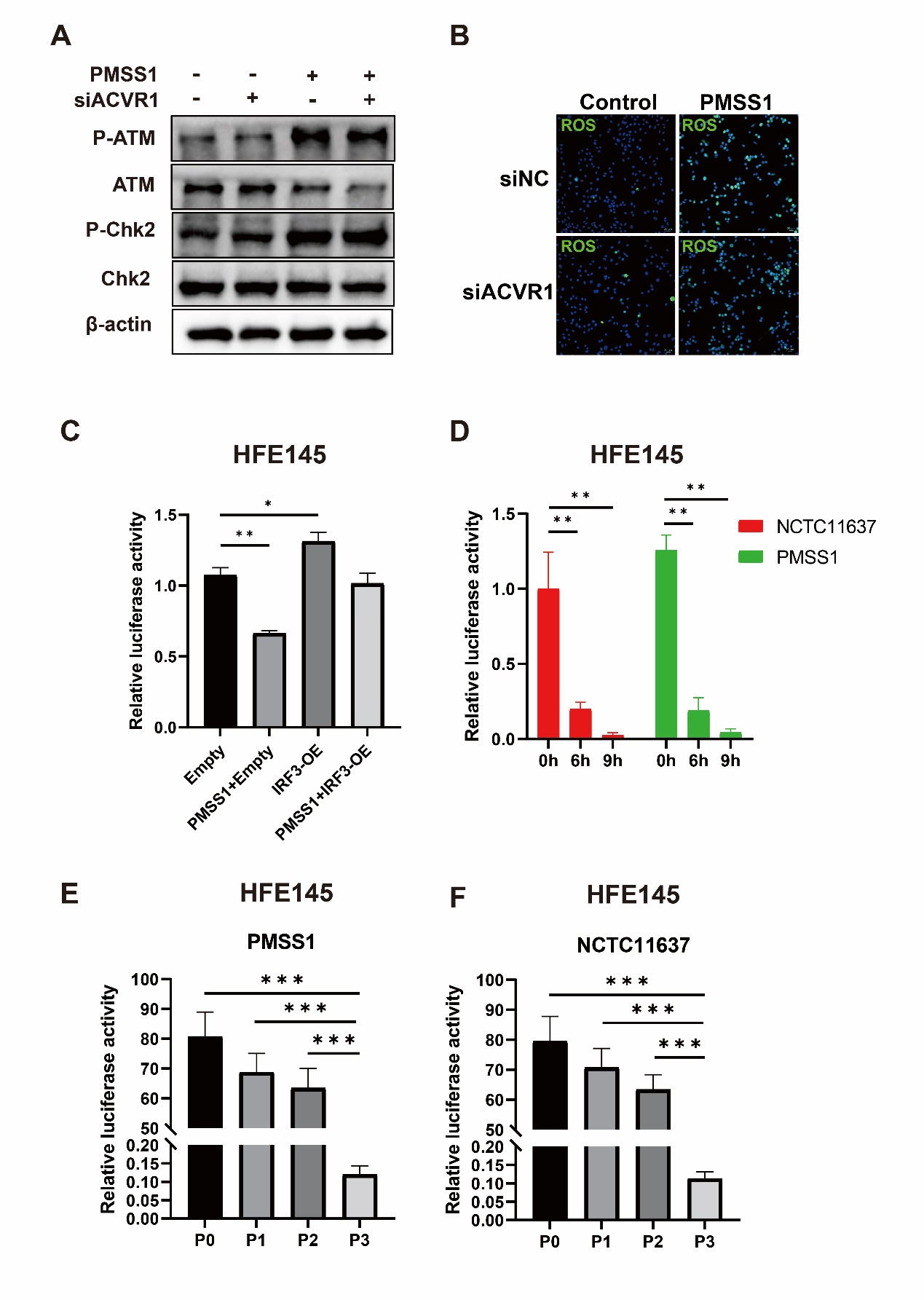


Figure S4


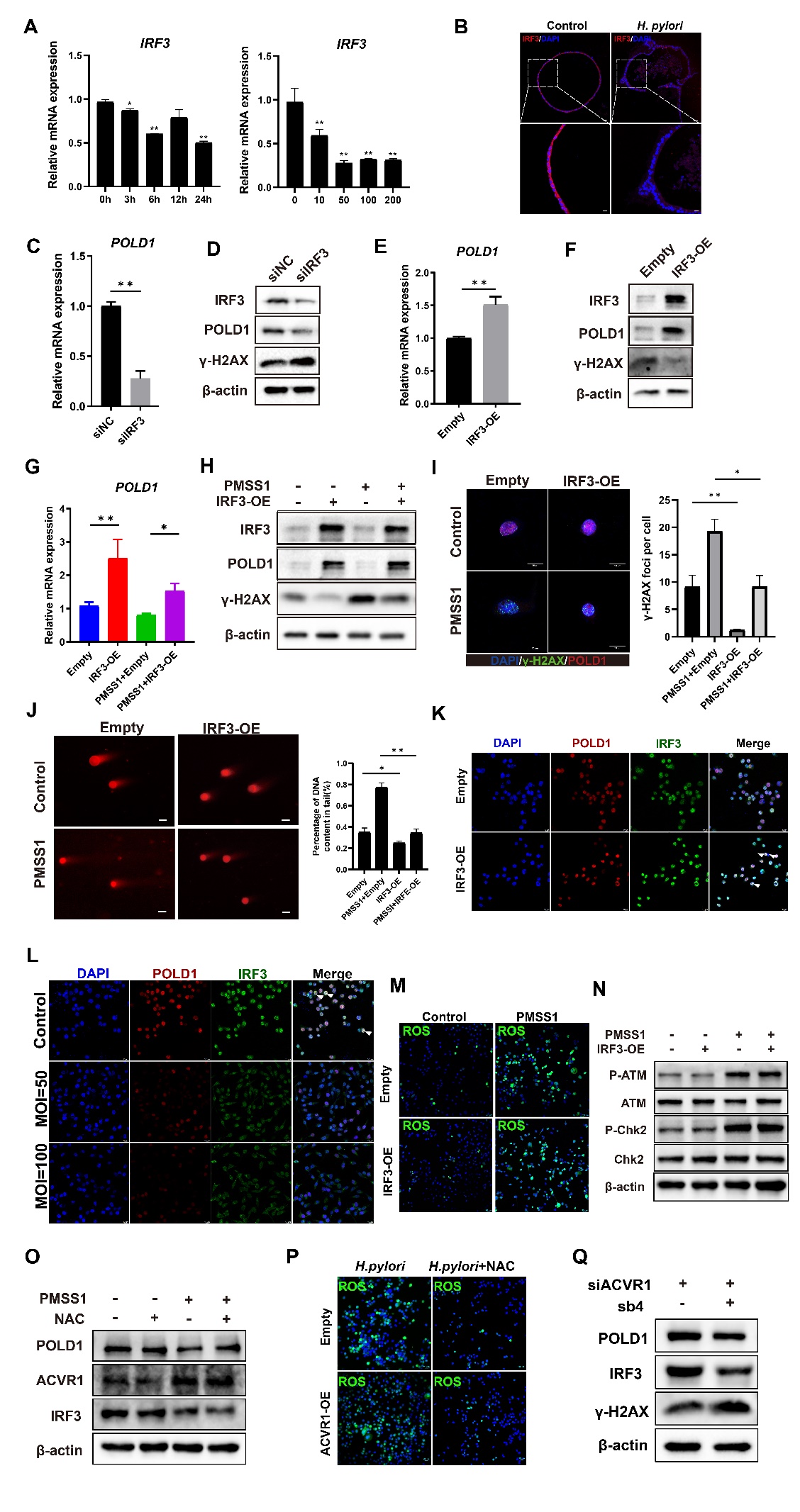


Figure S5


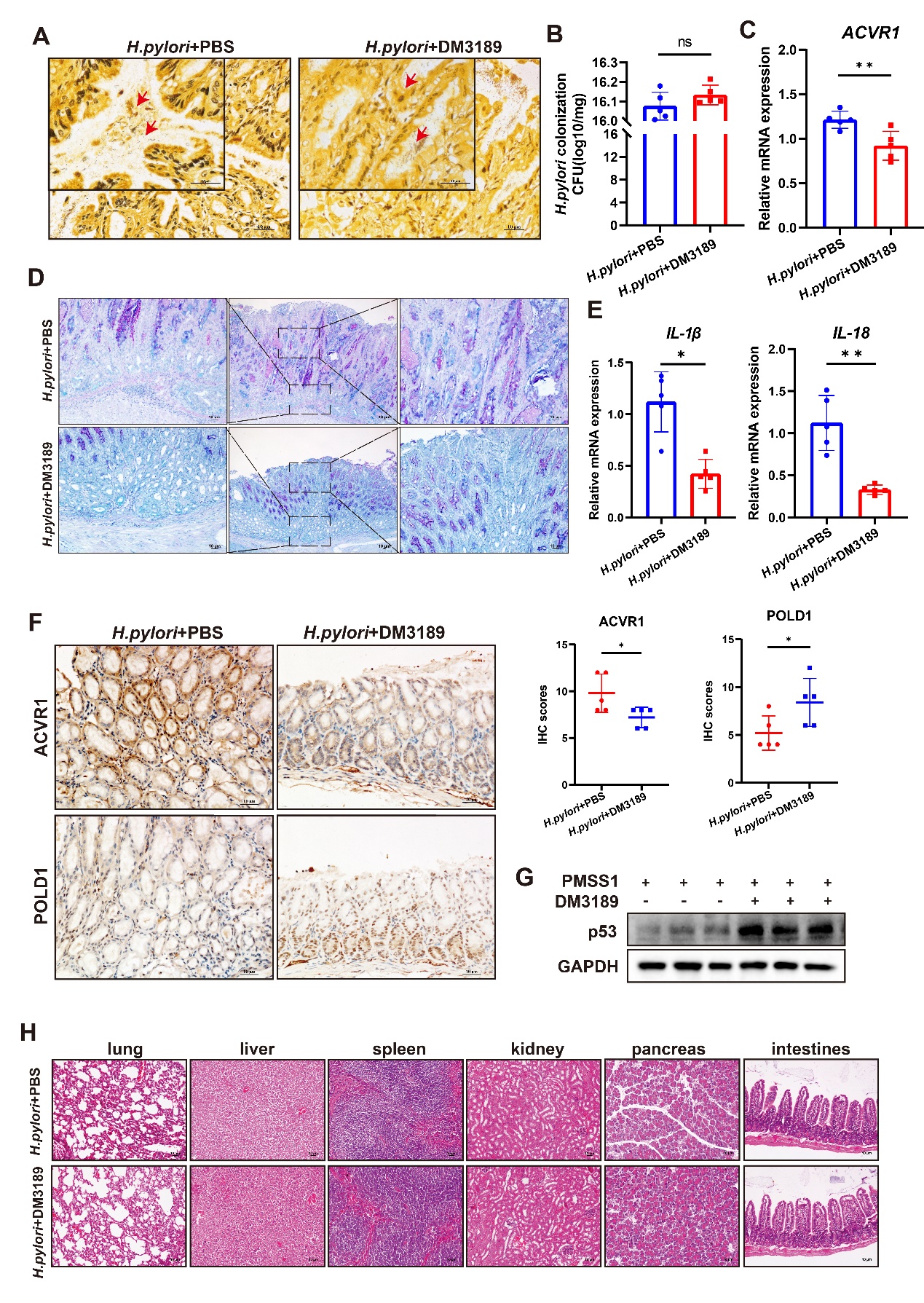


Figure S6

Supplement: Supplemental Material [file KGMI_A_2463581_SM8674.zip › Supp Figs.docx]
